# Re-evaluating the Potential Impact of Preexposure Prophylaxis (PrEP) in Achieving HIV Elimination in the United States: Insights From Modeling

**DOI:** 10.1093/ofid/ofag118

**Published:** 2026-03-04

**Authors:** Jason Baron, Tamar Tchelidze, Neil Parkin, Nicole Robinson, Benjamin La Brot, Aniruddha Hazra, Kenneth H Mayer

**Affiliations:** Roche Diagnostics, Medical and Scientific Affairs, Indianapolis, Indiana, USA; Roche Diagnostics, Medical and Scientific Affairs, Indianapolis, Indiana, USA; Roche Diagnostics, Scientific Communications, Sebastopol, California, USA; Roche Diagnostics, Medical and Scientific Affairs, Indianapolis, Indiana, USA; Roche Diagnostics, Medical Affairs, Pleasanton, California, USA; Section of Infectious Diseases and Global Health, Department of Medicine, University of Chicago, Chicago, Illinois, USA; Department of Medicine, Beth Israel Deaconess Medical Center, Harvard Medical School, The Fenway Institute, Fenway Health, Boston, Massachusetts, USA

**Keywords:** HIV, PrEP, modeling, risk, HIV population risk

## Abstract

**Background:**

Preexposure prophylaxis (PrEP) to prevent HIV-1 acquisition is a critical component of the strategy to achieve epidemic control and elimination goals. Previous studies assessing PrEP needs have not considered people based on individualized, quantitative risk profiles, potentially overlooking opportunities to increase PrEP use among all individuals vulnerable to acquiring HIV.

**Method:**

We modeled the risk distribution of sexually acquired HIV infection based on patterns of sexual activity among sexually active adults living in the United States. We estimated the population impact of PrEP use assuming “optimal” (PrEP perfectly distributed to those at highest risk) or “suboptimal” (distributed to only 50% of highest-risk individuals) PrEP distribution.

**Results:**

The model predicts that PrEP use by 1 million and 10 million individuals would prevent ∼3400 and 13 600 sexually acquired HIV infections per year, respectively, with optimal distribution, and 2700 and 9100 infections with suboptimal distribution. In addition to men who have sex with men, heterosexual women make up a significant proportion of the optimally targeted population.

**Conclusions:**

Our model provides a framework to estimate the potential population impact of PrEP. While results vary depending on assumptions, our findings strongly suggest that increases in PrEP uptake, substantially beyond current targets, may be required to achieve epidemic elimination goals. Our findings can inform updated assessments of PrEP coverage and provider initiatives at the individual level. Assessment of HIV acquisition risk based on individual behavior rather than risk-group membership indicates the imperative for wider PrEP distribution than currently occurs.

The US Department of Health and Human Services has set an ambitious goal to end the HIV epidemic in the United States by 2030, defined as a 90% reduction in new infections and diagnoses compared with the 2017 baseline (37 000 new infections and 38 531 new diagnoses per year) [1, 2]. The strategy to end the epidemic focuses on prevention, treatment, and addressing HIV-related disparities and health inequities [3]. While HIV incidence is declining, in 2022, an estimated 31 800 persons acquired HIV-1, representing a 14% reduction compared with the 2017 baseline, while new diagnoses decreased only 2% over the same period [4]. Achieving the goal of a 90% reduction in new infections corresponds to prevention of 33 300 new infections annually.

One of the most effective HIV-1 prevention modalities for those at risk of HIV acquisition is preexposure prophylaxis (PrEP) using antiretroviral agents, including oral administration of tenofovir disoproxil fumarate or tenofovir alafenamide in combination with emtricitabine [5, 6] or injectable, long-acting cabotegravir [7, 8] or lenacapavir [9, 10]. When taken as prescribed, PrEP has been demonstrated to be >99% effective and represents a powerful tool in the armamentarium for HIV-1 prevention [11–13]. To maximize the impact of PrEP and minimize unnecessary costs (including diagnostic testing) and toxicity, it is important to identify and prioritize PrEP for those most likely to benefit from it.

Certain types of behavior can increase the likelihood of HIV-1 transmission, including not using a condom during anal intercourse (especially for the receptive partner), and intravenous drug use with shared needles [14]. HIV-1 can also be transmitted via vaginal intercourse, albeit with lower probability. This has led to the US Centers for Disease Control and Prevention's (CDC) recommendation that all sexually active individuals should be informed about and offered PrEP [15]. Similarly, European and international guidelines recommend that PrEP be prioritized for individuals at substantial risk of HIV infection but also offered to anyone who requests it [16, 17].

Estimation of the number of individuals who are likely to benefit from PrEP is an essential undertaking for several reasons. “PrEP coverage,” representing the proportion of individuals who are likely to benefit from PrEP who are on PrEP, is a common metric to evaluate PrEP access programs. Changes in population-level incidence in relation to PrEP coverage is one indicator of real-world PrEP effectiveness [13]. Manufacturers and distributors of antiretroviral agents used for PrEP, and providers of diagnostic tests used to avoid PrEP initiation during unrecognized acute infection, can use this number to guide medication and assay production forecasting.

In 2015, the CDC estimated the number of individuals who could benefit from PrEP using national survey data [18]. The CDC classified survey respondents as to their need for PrEP using broad rules (eg, reporting 2 or more partners in the past year and recent condomless sex) to estimate the number of individuals who could benefit from PrEP by group (men who have sex with men [MSM], male and female heterosexually active adults (HAAs), and people who inject drugs [PWIDs]). This study concluded that 1.2 million people in the United States could benefit from PrEP. In 2025, the CDC and others updated this number under the presumption that individuals with an HIV risk of at least 1% could benefit from PrEP, by estimating the proportion of people in 4 groups (MSM, male and female HAA, and PWID) who have had an annual risk of incident HIV of 1% or greater [19]. This updated approach estimated that 2.3 million Americans could benefit from PrEP.

However, the focus on high-risk groups, even with an estimate of the size of subgroups at the highest risk, can potentially lead to perpetuating stigmas and a false sense of security among people identifying with a group defined as having a low overall risk, such as HAA men or women [20]. It can also result in a gross underestimation of individual risk profiles by patients and providers, as well as population-level underestimations of the number of people who could benefit from HIV screening and protection by PrEP. Moreover, the 2025 estimate of who could benefit from PrEP did not account for recent behavioral trends. There is growing evidence that the frequency of condomless anal sex has increased in recent years. For example, the 2015 estimate assumed that only 25% of MSM are at sufficient risk to benefit from PrEP; current estimates suggest that as many as 72% of all MSM contacts engage in condomless anal sex [21]. Prevalence of condomless anal intercourse (CAI) in 2018 was estimated to be 68% of men with HIV and 40% of HIV-negative men, and the proportion of men reporting never using a condom also increased from 2002 to 2018, almost doubling for HIV-negative men (from 6% to 11%) and tripling for men with HIV (from 7% to 21%) [22].

The 2015 CDC estimate assumed that only 0.4% of HAA were at risk, which is at odds with data that suggest much higher levels of CAI. One study reported that 48% of men and 38% of women had engaged in CAI in the past year [23].

To estimate the need for PrEP in the United States using a framework that quantitatively assesses individual HIV risk based on behavior without regard to specific group identity, we generated a model that describes the relationship between the number of people using PrEP and the number of HIV infections avoided.

## METHODS

We developed a model to estimate individuals' future risk of contracting HIV through sexual contact based on their anticipated sexual activity. We then applied this framework to National Health and Nutrition Examination Surveys (NHANES) respondents, using responses to survey questions about past sexual activity as a proxy for future sexual activity and taking the surveyed population as a representative sample of the US population in the relevant age ranges.

### Data Sources and Modeling

**Figure 1.**
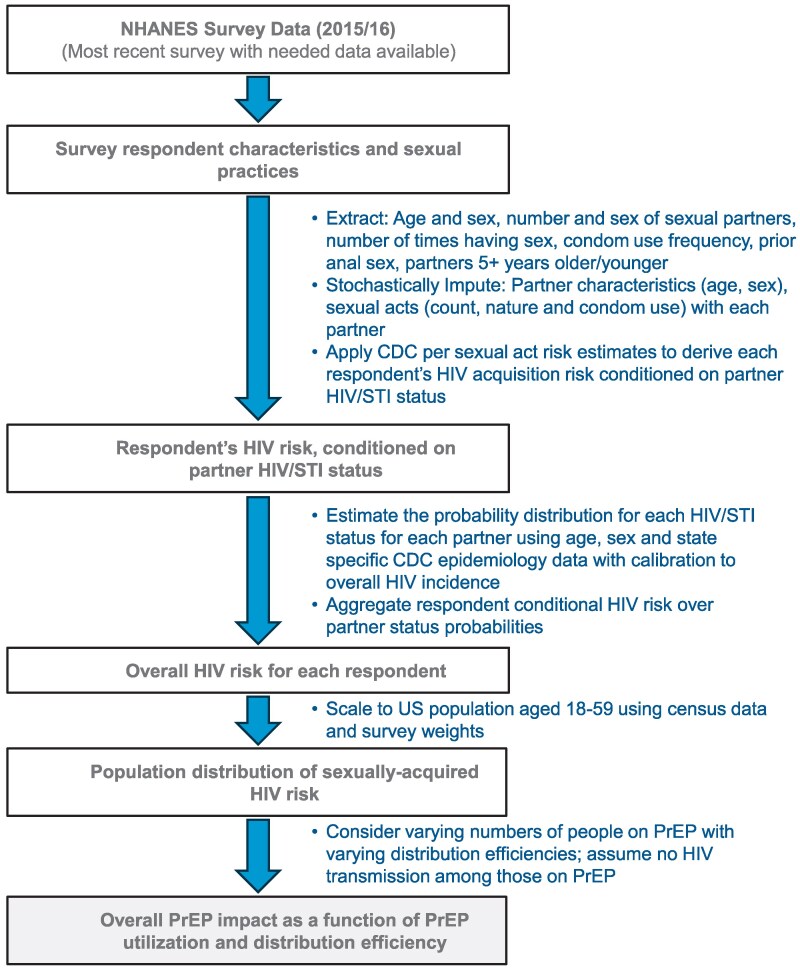
Overview of methods. We ran the simulation model described in the flowchart 200 times, bootstrapping survey responses. Odds of a survey respondent's sexual partner with HIV are assumed to be proportional to prevalence according to the partner's age, sex, and state. In our primary analysis, we assumed that partner risk is not correlated with respondent sexual practices. PrEP distribution efficiency: 100% means all individuals at highest risk receive PrEP until the available number of prescriptions is exhausted; 50% means the number of prescriptions is spread across twice as many individuals at highest risk (eg, 1 million prescriptions for 2 million individuals at highest risk). NHANES, National Health and Nutrition Examination Surveys; CDC, US Centers for Disease Control and Prevention; PrEP, preexposure prophylaxis.

An overview of the data sources and steps taken to develop the model is shown in Figure 1. We used the most recent (as of December 2024) NHANES available that included sexual health data (2015–2016) [24] to estimate HIV acquisition risk and the impact of PrEP at various levels of adoption and distribution efficiency.

Our model included 5 steps as detailed in the Supplementary material. We first extracted and imputed sexual practices for survey respondents. The survey data includes respondent biologic sex, the number and sex of sexual partners, the total number of sexual encounters with vaginal or anal intercourse, the frequency of condom use, and whether the respondent had previously engaged in anal sex. From this, we stochastically imputed the number and nature of specific acts with each partner (for each partner, the number of times having each of the following: vaginal receptive, vaginal penetrative, and anal receptive and anal penetrative intercourse with condoms and without condoms). We also stochastically imputed the US state. Second, we estimated the risk of HIV acquisition from each sexual partner of each respondent, conditioned on partner HIV and sexually transmitted infection (STI) status (Table 1; per sexual act, HIV transmission risk was derived from literature [14, 25–28] as described in the footnotes of Supplementary Table 1). Third, we estimated the likelihood of each STI and HIV status for each sexual partner. We estimated this based on HIV and STI prevalence among people of the partner's age, sex, and US state and a calibration factor derived from overall HIV incidence. In some cases, we also assumed a correlation between survey respondent sexual behavior and their partners' likelihoods of HIV. Fourth, we calculated each respondent's overall HIV risk and scaled the survey respondents to the US population (using survey weights) to calculate a population risk distribution. Fifth, we estimated the impact of PrEP under various distribution efficiencies by assuming that an individual in the population with an HIV risk of *P* (where *P* is a probability between 0 and 1) would be expected to prevent *P* HIV cases by taking PrEP (assumes 100% PrEP efficacy). We sum the values of *P* for all individuals in the population on PrEP to estimate the overall number of HIV cases avoided. We used 2 PrEP distribution scenarios: “optimal,” where PrEP is perfectly distributed to those at highest risk of HIV acquisition, and “suboptimal,” where N PrEP prescriptions are distributed evenly across the 2N highest risk people.

**Table 1.** HIV Risk Factors

Encounter-Type Factors^a^	Condom Use	Risk^b^
Anal insertive	Yes	0.0004
	No	0.0011
Anal receptive	Yes	0.0039
	No	0.0138
Vaginal insertive	Yes	0.0001
	No	0.0004
Vaginal receptive	Yes	0.0002
	No	0.0008
**Partner HIV Status**	**Partner STI Status**	**Risk Adjustment Factor** ^c,d^
Negative or undetectable viral load	Any	0
Positive with detectable viral load, not acute HIV	No STI	1
	STI	2.58
Acute HIV	No STI	7.25
	STI	18.705

See Supplementary Table 2 for estimate sources.

Abbreviation: STI, sexually transmitted infection.

^a^Receptive versus insertive from the perspective of the survey respondent.

^b^Risk of infection per act.

^c^The base per-act risk listed above is multiplied by this factor.

^d^These numbers are multiplied by 2.65 if the individual at risk for HIV has an STI.

### Model Assumptions

Detailed assumptions are provided in the supplemental methods. Key assumptions in our primary analysis include the following: (1) PrEP efficacy is 100% (derived from literature showing >99% efficacy [11–13]; we evaluated this assumption in sensitivity analysis); (2) in heterosexual individuals reporting anal intercourse, 10% of encounters involving intercourse on average include anal intercourse (this assumption was arbitrary but was tested robustly in sensitivity analysis); (3) there is either no or limited correlation between respondent sexual behavior and partner HIV risk, but relax this in sensitivity analysis; and (4) partner HIV likelihood is related to HIV prevalence among people of the partner's age, sex, and US state but is not affected by local admixture or sexual orientation (this assumption is made for simplification but likely leads to an underestimation of the heterogeneity in partner HIV risk; we partly evaluate the local admixture assumption in sensitivity analysis).

### Model Implementation

We implemented models in the R statistical scripting language (https://www.r-project.org/). We replicated the model 200 times for each set of parameters; with each replicate, we resampled (with replacement) survey respondents and re-imputed all stochastically imputed data elements. A detailed mathematical framework underpinning our model and a description of model parameters, assumptions, and additional data sources is included in the Supplementary material.

### Brief Intuition

Our approach estimated the distribution and heterogeneity of HIV acquisition risk via sexual exposure among individuals in the United States. High heterogeneity in risk among individuals (and thus concentrated risk among fewer individuals) implies that fewer individuals would need to be given PrEP to achieve a given level of HIV reduction. Consider a hypothetical population of 10 000 people with an HIV incidence of 10 cases (0.1%) per year: at one extreme of risk homogeneity, whereby everyone in the population has an equal risk of acquiring HIV, half the population (5000 individuals) would need to be given PrEP to achieve a 50% reduction in HIV. At the other extreme, if just 10 people have a 100% chance of acquiring HIV, 100% elimination could be achieved by providing PrEP to just those 10 individuals. While neither extreme is representative of reality, analyzing the behavioral risk spectrum of the US population can offer a window into the theoretical potential of PrEP.

## RESULTS

**Figure 2.**
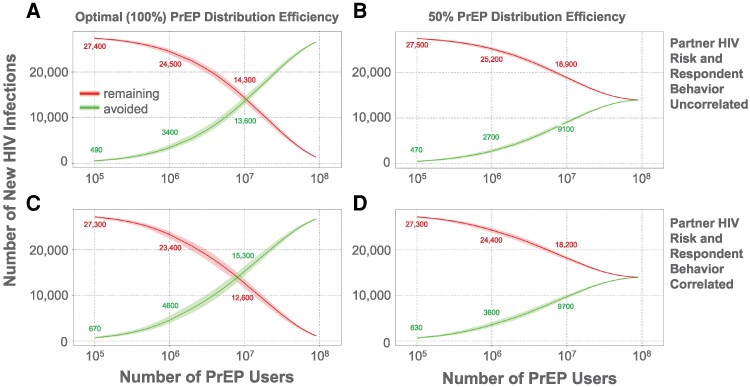
Predicted effects of preexposure prophylaxis (PrEP) adoption on the number of new HIV infections. The predicted number of new HIV infections annually (red, downward sloping curves) and infections avoided (green, upward sloping curves) are shown as a function of the number of people in the United States on PrEP. (*A* and *B*) Partner HIV risk and respondent behavior uncorrelated. (*C* and *D*) Partner HIV risk and respondent behavior correlated. (*A* and *C*) Optimal (100%) PrEP distribution efficiency; (*B* and *D*) 50% PrEP distribution efficiency.

The modeled cohort included 2927 survey respondents (1488 male and 1439 female) (Table 2). The relationship between the number of people using PrEP and the number of HIV infections averted or remaining per year is shown in Figure 2. Using default parameters with optimal PrEP distribution efficiency, 1 million and 10 million people using PrEP would result in prevention of a median of 3400 (10th–90th percentile: 2900–4100) or 13 600 (12 900–14 600) HIV infections per year, respectively (Figure 2*A*); with 50% distribution efficiency, 1 million or 10 million PrEP users would result in prevention of a median of 2700 (2400–3200) or 9100 (8800–9500) HIV infections per year, respectively (Figure 2*B*). (Optimal distribution efficiency means PrEP is distributed perfectly to those at highest risk, and 50% efficiency means 50% of highest-risk individuals get PrEP; see Methods.) When we assumed that respondents' sexual practices and their partner's likelihood of having HIV were correlated (λ_BehaviorRisk = 1, with λ_BehaviorRisk as defined in the Supplementary material), PrEP effectiveness improved (Figure 2*C* and 2*D*). The relationship between the threshold HIV risk (over 1 year) at which PrEP is given and percentage reduction in HIV transmission at the population level is shown in Supplementary Figure 1.

**Table 2.** Demographics of the Modeled Population

				Female						Male								
	All			HAA			Inactive			HAA			MSM			Inactive		
Age Group	N	%	W%	N	%	W%	N	%	W%	N	%	W%	N	%	W%	N	%	W%
18–31	1070	36.6	34.4	507	17.3	15.9	16	0.5	0.4	428	14.6	14.8	15	0.5	0.6	104	3.6	2.7
32–45	1027	35.1	33.3	514	17.6	16.6	7	0.2	0.2	477	16.3	16	2	0.1	0	27	0.9	0.5
46–59	830	28.4	32.3	389	13.3	15.1	6	0.2	0.2	386	13.2	14.8	9	0.3	0.7	40	1.4	1.6
All	2927	100	100	1410	48.2	47.6	29	1	0.8	1291	44.1	45.6	26	0.9	1.3	171	5.8	4.8

Abbreviations: HAA, heterosexually active adult; Inactive, sexually inactive; MSM, men who have sex with men; W%, weighted percent.

**Figure 3.**
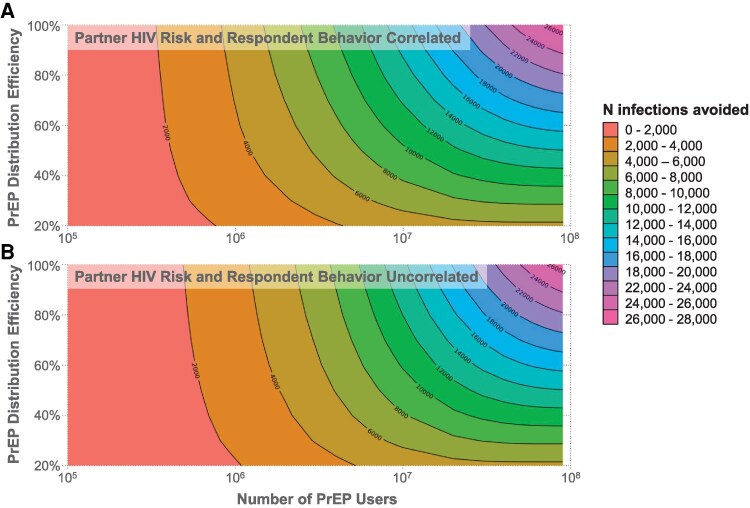
Predicted effects of preexposure prophylaxis (PrEP) adoption and distribution efficiency on the number of new HIV infections avoided. Color and contours show the number of new HIV infections avoided as a function of the number of people in the United States in PrEP (*x*-axis) and distribution efficiency (*y*-axis). (*A*) Partner HIV risk and respondent behavior uncorrelated. (*B*) Partner HIV risk and respondent behavior correlated.

The relationship between PrEP distribution efficiency, PrEP adoption rate, and the number of HIV-1 infections averted is shown in Figure 3; these data generalize the findings from Figure 2 to a range of distribution efficiencies. A similar relationship was observed for the number needed to treat per infection averted (Supplementary Figure 2). Although our primary analyses assumed perfect PrEP efficacy, model predictions under alternative efficacy values were also assessed (Supplementary Figure 3). The number of HIV cases avoided is proportional to one minus the efficacy (eg, 90% efficacy reduces cases avoided by 10% relative to 100% efficacy).

**Figure 4.**
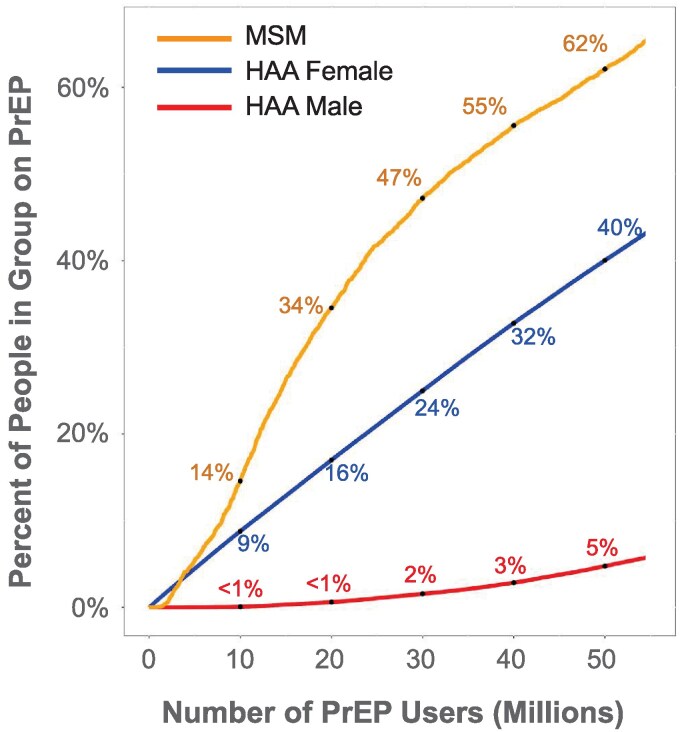
Prediction of the percentage of people in different groups on PrEP, assuming optimal (100%) PrEP distribution efficiency. MSM, men having sex with men; HAAs, heterosexually active adults; PrEP, preexposure prophylaxis.

Our model predicts the proportion of different groups that would receive PrEP given different total numbers of PrEP recipients. For example, under the assumption that PrEP distribution was optimal, if 10 million people were on PrEP, 14% of MSM, 9% of female HAA, and <1% of male HAA would be using PrEP (Figure 4). Similarly, Supplementary Table 4 shows the estimated number of individuals within each group who would need to be on PrEP to achieve various HIV reduction targets. To achieve a 50% reduction in population HIV incidence at optimal distribution, 979 000 MSM, 292 000 HAA males, and 12 035 000 HAA females may need PrEP (values listed here represent 90th percentiles across simulations; see Supplementary Table 4 for a wider percentile range). Because MSM individuals were likely undersampled in the NHANES, we also performed an additional analysis that upweights MSM respondents by a factor of 2 (see Supplementary Table 4). Illustrative examples of individuals who may need PrEP are shown in Supplementary Table 5.

We performed sensitivity analyses to determine the extent to which the model estimates are affected by variation in several parameters, including the degree of correlation between sex partners of survey respondents HIV and STI infection status (Supplementary Figure 4), probability of anal intercourse among HAA reporting it (Supplementary Figure 5), varying degrees of association between survey respondents' sexual risk behavior and their partners' HIV-1 infection status (Supplementary Figure 6), heterogeneity in number of sexual encounters (Supplementary Figure 7), probability of insertive or receptive anal sex in MSM (Supplementary Figure 8), and distribution of age difference between survey respondents and their sexual partners (Supplementary Figure 9). In these sensitivity analyses, only the degree of association between survey respondents' sexual risk behavior and their partners' HIV-1 infection status had a significant effect on the number of infections prevented at a given level of PrEP adoption and distribution efficiency. In addition, we performed a sensitivity analysis to evaluate how the model's predictions would vary if there were admixing subpopulations with an HIV prevalence much higher or lower than the state-wide averages used in our base case (Supplementary Figure 10). This analysis predicts that the greater extent to which there are subpopulations with HIV prevalence much higher than the average (ie, greater heterogeneity in HIV prevalence), the more impactful PrEP is likely to be at a given level of PrEP adoption and distribution efficiency.

## DISCUSSION

We have developed a behavior-based framework to estimate individuals' risk of future HIV acquisition through sexual transmission. By applying this framework to a sample of the US population (NHANES respondents), we estimated the distribution of HIV acquisition risk across the United States and the potential impact of PrEP. We found that PrEP adoption on the order of 10 million individuals or more may be needed to achieve reductions in sexually transmitted HIV infections of at least 50%. This contrasts to previous CDC estimates of 1.2 million [18] and 2.3 million [19] individuals needing PrEP. We also find that a much larger proportion of heterosexual females require PrEP compared to prior estimates.

We acknowledge that this study does not itself offer new, rule-based guidance around who should get PrEP. Indeed, because the model quantitatively accounts for each sexual encounter, model outputs cannot be reduced to a simple set of rules; translating our model to practice would require development of clinical calculators or decision support systems (in addition to significant additional validation). Nonetheless, the results offer strong evidence that existing PrEP uptake will need to increase substantially beyond current targets to achieve HIV elimination goals. While PrEP is not the only tool for HIV prevention, it is arguably critical to achieving HIV elimination goals.

Our approach and findings offer several important insights. By focusing on sexual behavior rather than group identity or reported sexual orientation, we may identify individuals who are vulnerable but belong to groups typically considered less affected. The results highlight that a substantial number of heterosexual women may engage in behaviors that put them at particularly high risk of HIV acquisition. While a wide range of specific behaviors and factors may contribute to cumulative HIV risk in heterosexual women, condomless, receptive, anal intercourse may be a substantial contributor in some cases.

Nonetheless, this study is subject to important limitations: (1) The NHANES data may not be fully representative of the US population. While we used survey weights to partly adjust for sampling bias, it appears that MSM individuals may remain underrepresented; other sampling biases are also possible. (2) NHANES data available lacked certain details, necessitating imputation (see Methods). This may have introduced imprecision or bias (though our stochastic approach to imputation should have partly mitigated this). (3) We lacked a definitive empirical basis to select a particular value for certain parameters; we used sensitivity analyses to confirm the robustness of findings across a range of values for these parameters. (4) Our approach to estimating partner HIV likelihood is imprecise and may underestimate heterogeneity in this parameter. If it does, it would tend to make PrEP appear to be less effective at a population level at any given level of adoption.

One important factor potentially causing our model to underestimate heterogeneity in partner HIV likelihood is that our core analysis does not account for higher HIV prevalence in subpopulations due to local admixing and does not adjust HIV prevalence for sexual orientation. For example, an MSM male partner was assumed to have an identical baseline risk to an HAA male. Likewise, our model would consider 2 individuals of the same age and sex and with identical patterns of sexual behavior to have equal risk even if one is living in a suburban and another in an urban area of the same state with very different background levels of HIV prevalence. In this case, it may be possible that one of the individuals would need PrEP while the other would not. We partly evaluate the local admixing limitation through sensitivity analysis. We do not adjust partner risk for sexual orientation, in part because one of our goals was to develop a risk model independent of sexual identity. However, not adjusting partner risk for sexual orientation may be causing our model to underestimate PrEP need among MSM individuals and overestimate them among heterosexual females. While clinicians can use the information from our model and other studies [19] to provide general insights, they must use broader information from the individual patient to make an informed decision about the appropriateness of PrEP. Overall, while these limitations are important, we believe that the value of our model is inherent in its framework and qualitative conclusions.

We note that our results represent a snapshot in time. Over time, we would expect a multiplier effect from increased PrEP usage, whereby HIV prevalence and thereby transmission rates decline. Our approach also does not account for potential changes to the PrEP landscape.

Our work provides a complementary perspective to the previously noted estimates of PrEP need published by Kourtis et al [19] (described in the Introduction) who estimated that a substantial proportion of incident HIV occurs in individuals with an HIV risk >1%. Instead, our model estimates that individuals with incident HIV typically do not exceed this HIV risk threshold (Supplementary Figure 1). This likely in part represents a limitation of our model (see above). Nonetheless, it also highlights the strong possibility that individuals with HIV risk substantially <1% may need to be offered PrEP to effectively achieve HIV elimination targets. Overall, given that any approach to estimating PrEP need will necessarily rely on multiple assumptions and estimations, we believe it is useful to synthesize multiple estimates derived using different methodologies.

Future research refining our approach and underlying assumptions would be useful. We also propose future studies involving agent-based models to better capture localized patterns of admixture and HIV risk.

We have undertaken this effort as part of our ongoing commitment toward the current goals of ending the HIV epidemic by 2030 and reducing stigma associated with the definition of “high-risk” groups based on externally defined characteristics instead of individual behaviors that may be undertaken by individuals in any demographic group. While specific findings will vary when applying our framework to alternative datasets, or including alternative assumptions, we anticipate that the general approach can serve as a foundation for future research attempting to better model HIV risk and PrEP utility.

## Supplementary Material

ofag118_Supplementary_Data

## Supplementary Data

Supplementary materials are available at *Open Forum Infectious Diseases* online. Consisting of data provided by the authors to benefit the reader, the posted materials are not copyedited and are the sole responsibility of the authors, so questions or comments should be addressed to the corresponding author.
