# Supplementary material for: Re-evaluating the Potential Impact of Preexposure Prophylaxis (PrEP) in Achieving HIV Elimination in the United States: Insights From Modeling
Source: Open Forum Infect Dis. 2026 Mar 4;13(3):ofag118. doi: 10.1093/ofid/ofag118 (PMC13006136; doi:10.1093/ofid/ofag118)
Supplement: ofag118_Supplementary_Data [file ofag118_supplementary_data.pdf]

ONLINE SUPPLEMENT

Re-evaluating the Potential Impact of PrEP in Achieving HIV Elimination in the United States: Insights from Modeling

Contents

SUPPLEMENTAL METHODS ... 3

General Intuition ... 3

Mathematical Framework ... 4

Step 1: NHANES Survey Data Extraction and Imputation ... 4

Table S1: Imputation of Sexual Encounters ... 5

Step 2: Estimate Of Respondent HIV Acquisition Risk From Each Sexual Partner Of Each Respondent, Conditioned On Partner HIV/ STI Status ... 6

Eq 1: Per Sexual Encounter Risk ... 6

Table S2: Per-Act HIV Transmission Risk ... 7

Eq 2: Respondent Conditional HIV Risk On An Encounter Basis. ... 8

Eq 3: Respondent Conditional HIV Risk On A Partner Basis. ... 8

Step 3: Estimate The Likelihood Of Each STI/HIV Status For Each Partner ... 8

Eq 4: Partner HIV/STI Status Likelihood ... 8

Eq 4a ... 8

Eq 4b ... 8

Step 4: Calculate each respondent's overall HIV risk and scale to the US population to calculate a population risk distribution ... 9

Step 4a: Overall HIV Risk By Respondent ... 9

Eq 5: Total Risk for Respondent Across Partners ... 9

Step 4b: Scale Survey Respondents to the US Population ... 9

Eq 6: Scaling Survey Respondents to the Population ... 9

Eq 6a ... 9

Eq 6b ... 9

Eq 6c ... 10

Eq 7: New HIV Cases In Population Scaled From Each Respondent ... 10

Eq 7a ... 10

Eq 7b ... 10

Step 5: Estimate The Impact Of Prep By Adoption And Distribution Efficiency ... 10

Eq 8a: Population at Risk Greater than a Given Survey Respondent ... 10

Eq 8b: Population of Potential PrEP Candidates 11
Table S3: Number Of People In The Population Represented By Survey Respondent i Who Will Receive Prep 11
Eq 9: Total HIV Cases Avoided by Survey Respondent 12
Eq 10: Total HIV Cases Avoided Across the Population 12
Parameter Values 13
US Population at Risk and Total Sexually Transmitted HIV Cases 14
Model Implementation 15
Additional Method Details 16
Calculating Distribution of Encounters across Partners 16
Sampling Partner Ages 16
Estimating Likelihood of Each Partner HIV/STI Status 17
Sensitivity Analysis Evaluating The Impact Of Localized Variation In HIV Prevalence 18
SUPPLEMENTAL RESULTS 19
Table S4: Predicted Number of PrEP Users by Group as a Function of HIV Reduction Goal 19
Table S5: Illustrative Examples of Individuals Who the Model Predicts May Need PrEP 20
Figure S1: Population HIV Reduction vs. Individual Risk Threshold at Which PrEP is Given 21
Figure S2: Number Needed to Treat as a Function of PrEP Adoption and Distribution Efficiency 22
Figure S3: Impact of Imperfect PrEP Efficacy 23
Figure S4: Sensitivity Analysis Examining the Impact of Introducing Correlation Between Partner HIV likelihood and STI likelihood 24
Figure S5: Sensitivity Analysis, Impact of varying rates of anal intercourse among HAA 25
Figure S6: Sensitivity Analysis, Impact Of Varying Correlation Between Partner HIV-1 Infection Status And Respondent Sexual Risk Behavior 26
Figure S7:. Sensitivity Analysis, Impact Of Varying Distribution Of Sexual Encounters Across Sexual Partners 27
Figure S8: Sensitivity Analysis, Impact Of Varying Distribution Of Receptive Vs. Insertive Intercourse Among MSM Individuals 28
Figure S9: Sensitivity Analysis, Impact Of Varying Distribution Of Age Difference Between Survey Respondents And Their Partners 29
Figure S10: Sensitivity Analysis, Impact Of Variation In HIV Prevalence Among Localized Subpopulations 30
.... **Error! Bookmark not defined.**

SUPPLEMENTAL METHODS

General Intuition

Fundamentally, we developed a model to estimate the distribution of HIV risk within the US population. From this distribution, and as described in greater detail below, we then estimated the theoretical impact of PrEP on HIV transmission at a given level of adoption and distribution efficiency. As mathematically described below, the distribution efficiency denotes the extent to which PrEP is given to the highest risk vs. lower risk individuals.

One component of our model seeks to estimate a single individual's risk of acquiring HIV through sexual contact over the coming year. This component of the model treats as fixed the individual's STI status and sexual behavior, including the number, age and sexes of the individual's sexual partners and the sexual acts performed with each partner (including the number of times each act is performed with the partner over the course of the year, the type of sexual act and whether a condom was used). While an individual deciding whether to go on PrEP may not know this information, we assume that this information can be estimated/predicted with sufficient accuracy when individuals are determining their risk for the purposes of deciding whether to go on PrEP.

Within this framework, we can estimate the individual risk of acquiring HIV on each sexual encounter conditioned on each partners' HIV status using previously established estimates (see below) of per sexual act HIV transmission risk (based on the nature of the act and condom use). We can aggregate these probabilities over all the partners and encounters for the individual to establish the individual's aggregate HIV risk conditioned on partner HIV/STI statuses.

We assume partner HIV statuses as unknown (paralleling the idea that an individual deciding whether to go on PrEP generally would not know with certainty the HIV statuses of the partners). However, we estimate a likelihood that each partner has HIV and/or an STI based on the partner's age, sex and state and in some cases, the individual's sexual activity; we further calibrate our model to set overall population partner HIV likelihood based on established national rates of HIV transmission.

By applying the probability chain rule and aggregating the conditional probabilities over all partner statuses, we can then estimate the individual's overall HIV risk.

After establishing a framework to estimate an individual's HIV risk over the course of a year, we apply this framework to all individuals within an NHANES survey to estimate the distribution of HIV risk across the population. We then consider, if all or subsets of the "N" highest risk individuals go on PrEP, how this will impact overall HIV transmission. The general premise is, for example, that if 100 individuals each with 1% chance of HIV go on PrEP, this will be expected to eliminate 1 HIV case. If 100 individuals with 2% chance of HIV go on PrEP, this will eliminate 2 cases and so forth.

Based on this intuition, we specifically developed a five step model as described mathematically below.

Mathematical Framework

Below is a mathematical description of the model divided into five "steps" for intuition.

Step 1: NHANES Survey Data Extraction and Imputation

We used data from the CDC's NHANES survey (2015/16, most recently available with needed sexual health data).

For each survey respondent 18-59, we extracted age, reported biologic sex, number of male and number of female sexual partners over the past year (for vaginal or anal sex), frequency of condom use over the past year, number of times having vaginal or anal intercourse over the past year (what we call "encounters"), any anal sex (lifetime) and survey weights.

While we know total sexual encounters and total partners, we cannot directly determine how many encounters were with each partner; we used a distribution function parameterized by a parameter $\lambda_{Encounter\_Heterogenity}$ that ranges from -100 to 100 where -100 indicates maximum homogeneity where all partners have an equal number of encounters (within 1) and 100 indicates maximum heterogeneity (all partners except 1 have only 1 encounter each with remaining encounters assigned to a single partner). We use -100 as a default but use other values in sensitivity analysis.

For each encounter, we need to impute the sexual acts performed (anal vs. vaginal; receptive vs insertive; condom use) Table S1. (We know from responses that encounters involve either vaginal or anal intercourse.) If the respondent answered no to ever having anal sex, any heterosexual encounters are assumed to be vaginal insertive (if the respondent is male) or vaginal receptive (if respondent is female). If the respondent answered yes to having anal sex, we handle heterosexual encounters similarly except we replace vaginal receptive or insertive with anal receptive or insertive for randomly selected encounters (selected with a probability denoted by a parameter λ_{HAA_Anal}). Homosexual encounters (defined by the questions as anal or vaginal intercourse) between men are either anal insertive or anal receptive. Each respondent engaging in homosexual encounters is imputed a variable (randomly sampled with 50% chance) whether the respondent has more receptive or insertive intercourse; we then sample each encounter as insertive or receptive with a probability of $\lambda_{\mathrm{MSM\_receptive}}$ if the respondent has more receptive intercourse or 1-$\lambda_{\mathrm{MSM\_receptive}}$ if the respondent has more insertive. Condom use for each encounter was sampled with a probability based on the respondent response to the survey question about condom use over the past year as shown in Table S1.

Table S1: Imputation of Sexual Encounters

Respondent Gender	Partner Gender	Respondent History of Anal Sex	Respondent Majority Receptive	Sex Act	Sampling Probability
Female	Male	No	NA	Vaginal receptive	1
		Yes		Vaginal receptive	1- λ_{HAA_Anal}
				Anal receptive	λ_{HAA_Anal}
Male	Female	No		Vaginal insertive	1
		Yes		Vaginal insertive	1-λ_{HAA_Anal}
				Anal insertive	λ_{HAA_Anal}
Male	Male	NA	Yes	Anal insertive	1- $\lambda_{\mathrm{MSM\_receptive}}$
				Anal receptive	$\lambda_{\mathrm{MSM\_receptive}}$
			No	Anal insertive	$\lambda_{\mathrm{MSM\_receptive}}$
				Anal receptive	1- $\lambda_{\mathrm{MSM\_receptive}}$

**Respondent Response regarding condom use over prior year**	**Sampling probability determining whether an encounter involved condom use**
Always	1
Not always, but more than half of the time	0.75
About half of the time	0.5
Less than half of the time	0.25
Never	0

- λ_{HAA_Anal} is a parameter for the proportion of heterosexual anal/vaginal intercourse among respondents who indicated a history of anal sex that is comprised of anal sex. Default=0.1
- For each MSM respondent we randomly assign at the respondent level an attribute (with 50:50 chance) indicating that the respondent engages primarily in receptive intercourse or primarily in insertive intercourse. (Yes or No in the fourth column). We then include a parameter $\lambda_{\mathrm{MSM\_receptive}}$; this parameter describes the proportion of MSM encounters that are insertive vs. receptive. If $\lambda_{\mathrm{MSM\_receptive}}$=0.5 (default value) the respondent majority receptive becomes irrelevant. If $\lambda_{\mathrm{MSM\_receptive}}$=1, a given MSM respondent will engage in either receptive or insertive intercourse but not both.

Respondent US states of residence were sampled with probabilities based on the proportion of the US population of the respondent's age and gender residing in each US state (Census data). Partner states were assumed to be the same as the respondent state. Respondent ages were assigned by sampling an age difference between the survey respondent and the partner; age differences were assumed to follow a normal distribution with means and standard deviations as discussed in the supplemental methods.

Step 2: Estimate Of Respondent HIV Acquisition Risk From Each Sexual Partner Of Each Respondent, Conditioned On Partner HIV/ STI Status

We first estimate the risk that each respondent will acquire HIV from each encounter. We use per act estimates as in Table S2 . The numbers in this table (and the individual STI factor shown) were originally derived from the CDC's Online HIV Calculator; this calculator is no longer available as of this writing, but similar (and in many cases identical) numbers can be found in Patel et al. (Patel et al. 2014. AIDS. 28(10): 1509–1519.)

Eq 1: Per Sexual Encounter Risk

$$risk_{i,j,k} = b_{i,j,k} * Individual.STI_i * \ Partner.Status_{i,j}$$

Where $risk_{i,j,k}$ is the risk of respondent *i* getting HIV from partner *j* on encounter *k*. $b_{i,j,k}$ is determined by the sexual act as shown in Table S2a. $Individual.STI_i$ is 2.65 if the individual has an STI and 1 otherwise. $Partner.Status_{i,j}$ is determined based on the partners STI/HIV status as shown inTable S2b. (The value of 2.65 is from Hughes JP, Baeten JM, Lingappa JR, et al. Determinants of per-coital-act HIV-1 infectivity among African HIV-1-serodiscordant couples. J Inf Dis 2012;205(3):358-65.)

Table S2: Per-Act HIV Transmission Risk

**Table S2a**: Encounter-type factors			
**Encounter$_{i,j,k}$**	**Condom Use**	**$b_{i,j,k}$**	**Source/ Calculation**
Anal insertive	Yes	0.0004	1,2,3
	No	0.0011	1
Anal receptive	Yes	0.0039	1,2,4
	No	0.0138	1
Vaginal insertive	Yes	0.0001	1,5,6
	No	0.0004	1
Vaginal receptive	Yes	0.0002	1,5,6
	No	0.0008	1

**Table S2b**: Partner-base factors			
**Partner HIV Status**	**Partner STI-Status**	$\boldsymbol{Partner.Status_{i,j}}$	**Source/ Calculation**
Negative or UVL	Any	0	Partner Non-infectious
Positive	No STI	1	Base case without adjustment
	STI	2.58	7
Acute HIV	No STI	7.25	8
	STI	18.705	9

Receptive vs. insertive from the perspective of the survey respondent (i). UVL= undetectable viral load. Positive = HIV positive with detectable viral load but not acute HIV

Sources/ Calculations:

1) Patel P, Borkowf CB, Brooks JT, et al. Estimating per-act HIV transmission risk: a systematic review. AIDS. 2014.
2) Smith DK et al. Condom effectiveness for HIV prevention by consistency of use among men who have sex with men in the United States. J Acquir Immune Defic Syndr. 2015;68:337-44.
3) The risk with condom use was calculated by applying a 63% risk reduction (rounded to two significant figures) from condom use based on reference 2 to the per act risks from reference 1 and rounding to four decimal places
4) The risk with condom use was calculated by applying a 72% risk reduction (rounded to two significant figures) from condom use based on reference 2 to the per act risks from reference 1 and rounding to four decimal places
5) Weller SC and David-Beaty K. Condom effectiveness in reducing heterosexual HIV transmission (Review). Cochrane Database Syst Rev 1. (2002): CD003255.
6) The risk with condom use was calculated by applying an 80% risk reduction from condom use based on reference 5 to the per act risks from reference 1 and rounding to four decimal places.
7) Gray RH, Wawer MJ, Brookmeyer R, et al. Probability of HIV-1 transmission per coital act in monogamous, heterosexual, HIV-1-discordant couples in Rakai, Uganda. Lancet 2001;357:1149-53.
8) Wawer MJ, Gray RH, Sewankambo NK, et al. Rates of HIV-1 transmission per coital act, by stage of HIV-1 infection, in Rakai, Uganda. J Infect Dis 2005;191:1403-9.
9) Calculated by multiplying the risk adjustment from reference 7 (2.58) by the risk adjustment from reference 8 (7.25)

Specific values selected to parallel previously available CDC web-based materials.

From this, we can determine each respondent's conditional HIV risk from each partner as shown in Eq 2. We assumed that HIV risk from other sexual acts (e.g. oral sex) was negligible and thus we did not consider these in our model.

Eq 2: Respondent Conditional HIV Risk On An Encounter Basis.

$$r_{i,j,k} = \sum_{All\ s} \mathrm{pr}(Partner\_Status_{i,j} = s) * \left(risk_{i,j,k} | Partner\_Status_{i,j} = s\right)$$

$r_{i,j,k}$ represents the risk that respondent *i* contracts HIV from partner j on encounter k. Where $\mathbf{pr}(\boldsymbol{Partner\_Status_{i,j}} = \boldsymbol{s})$ is the probability that **partner *j*** (of respondent *i*) has HIV/STI status ***s,*** as defined in Table S2b. $risk_{i,j,k}|Partner\_Status_{i,j} = s$ is the encounter risk as defined in Eq 1 conditioned on the **partner *j*** (of respondent *i*) having HIV/STI status ***s.*** (This equation is derived from the conditional probability chain rule). Here ***s*** includes all of the statuses shown in Table S2b.

We aggregate across all encounters between respondent i and partner j as in Eq 3.

Eq 3: Respondent Conditional HIV Risk On A Partner Basis.

$$R_{i,j} = 1 - \left[\prod_{all\ k} \left(1 - r_{i,j,k}\right)\right]$$

Where $R_{i,j}$ is the aggregate risk that respondent i acquires HIV from partner j and $r_{i,j,k}$ is as in Eq 2.

Step 3: Estimate The Likelihood Of Each STI/HIV Status For Each Partner

Here we define the likelihood of each partner having HIV or an STI. We base this on a combination of factors including the prevalence of HIV and chlamydia among people of the partner's age, sex and state. In some scenarios, we also include a factor that assumes that on average, respondents engaging in riskier sexual practices also have partners that are more likely to have HIV or STIs as shown in **Eq 4.**

Eq 4: Partner HIV/STI Status Likelihood

Eq 4a

$$\boldsymbol{ln}\left(odds_{i,j,s}\right) = \boldsymbol{ln}\left(prev.odds_{i,j,s}\right) + \lambda_{BehaviorRisk} * \sum_{All\ k} b_{i,j,k} + \lambda_{Status_{Adjustor}}$$
$$for\ s \notin HIV_{Neg}$$

Where $\boldsymbol{ln}\,(odds_{i,j,s})$ is equivalent to $\mathrm{pr}(Partner\_Status_{i,j} = s)$ in Eq 2 when expressed as a log-odds. $\boldsymbol{ln}\left(prev.odds_{i,j,s}\right)$ is the prevalence of the HIV/STI status for people of the partner's age, sex and state (expressed as a log-odds). We estimate $prev.odds_{i,j,s}$ from CDC data as shown in the supplemental appendix. $\sum_{All\ k\ for\ i\ and\ j} b_{i,j,k}$ is the sum over all encounters (k) between respondent i and partner j of $b_{i,j,k}$, with $b_{i,j,k}$ as defined in Eq 1. $\lambda_{BehaviorRisk}$ is a parameter which we vary with a value of 0 in our primary analysis (suggesting that partner HIV likelihood is independent of the individual's sexual practices. $\lambda_{Status_{Adjustor}}$ is a calibration constant as discussed later. To combine Eq 2 with Eq 3, we can convert the log odds into a probability with Eq 4b.

Eq 4b

$$\mathrm{pr}\left(Partner_{Status_{i,j}} = s\right) = \frac{e^{\boldsymbol{ln}\,(odds_{i,j,s})}}{1 + e^{\boldsymbol{ln}\,(odds_{i,j,s})}}$$

In theory $\text{pr}\left(Partner_{Status_{i,j}} = s\right)$ for the status of HIV negative could be calculated as 1 minus the sum of the probabilities of other cases; however, in implementation, it is not necessary to calculate this since this contributes 0 transmission risk.

Step 4: Calculate each respondent's overall HIV risk and scale to the US population to calculate a population risk distribution

Step 4a: Overall HIV Risk By Respondent

We can aggregate Eq 3 across all partners as in Eq 5.

Eq 5: Total Risk for Respondent Across Partners

$$Total.Risk_i = 1 - \left[\prod_{all\ j}\left(1 - R_{i,j}\right)\right]$$

Where $Total.Risk_i$ is the aggregate risk that respondent i acquires HIV across all partners and $R_{i,j}$ is as in Eq 4**.**

Step 4b: Scale Survey Respondents to the US Population

We assume that each survey respondent scales to a given number of people in the overall US population. We use the terminology "population represented by survey respondent $\boldsymbol{i}$" to represent the theoretical people in the US population corresponding to survey respondent $\boldsymbol{i}$ when scaled.

We mathematically scale to the US population based on survey weights as in Eq 6.

Eq 6: Scaling Survey Respondents to the Population

Eq 6a

$$Total.Weights = \sum_{i=1}^{N.respondents} W_i$$

Where $Total.Weights$ is the total survey weights of all respondents included in the analysis. W_i represents the survey weight associated with respondent $\boldsymbol{i}$. Survey weights are taken directly from the survey data and are inversely proportional to the likelihood that a survey respondent will be sampled. In the complete survey, weights sum roughly to the US population. However, because only a subset of individuals could be included in our analysis, we scale accordingly as below.

Eq 6b

$$\Phi = \left(\frac{P_{overall}}{Total.Weights}\right)$$

Where Φ is a scaling factor indicating the number of people corresponding to a unit of survey weight. $P_{overall}$ represents the overall US population within the relevant age range (US Census Bureau Data, see below.) $Total.Weights$ is as in Eq 6a.

Eq 6c

$$P_i = W_i * \Phi$$

Where P_i represents the relevant US population to which survey respondent $\boldsymbol{i}$ scales. W_i represents the survey weight associated with respondent $\boldsymbol{i}$. Φ is as in Eq 6b.

In the absence of PrEP, we can estimate the number of new HIV transmissions among the population represented by each respondent as in Eq 7.

Eq 7: New HIV Cases In Population Scaled From Each Respondent

Eq 7a

$$HIV_i = P_i * Total.Risk_i$$

Where HIV_i represents number of HIV cases in the US population scaled from respondent $\boldsymbol{i}$**,** and P_i is as in Eq 6 and $Total.Risk_i$ is as in Eq 5**.** Summing Eq 7a over all respondents will allow us to estimate total HIV cases from sexual transmission as shown in Eq 7b.

Eq 7b

$$HIV_{Total} = \sum_{i=1}^{N.respondents} HIV_i$$

Where HIV_{Total} is total HIV incidence (sexually acquired in the relevant age ranges) in the absence of PrEP.

We empirically set $\lambda_{Status_{Adjustor}}$ such that HIV_{Total} matches the total HIV incidence (CDC data, see below).

Step 5: Estimate The Impact Of Prep By Adoption And Distribution Efficiency

Conceptually, assume that we rank all survey respondents by estimated HIV risk. We then distribute PrEP to a given percentage (PrEP distribution efficiency denoted by $\lambda_{Efficiency}$) of the US population scaled from each survey respondent in order of risk until N_{PrEP} total PrEP prescriptions are distributed (at which point the population scaled from additional respondents do not receive PrEP).

Mathematically, we can represent this as in Eq 8:

Eq 8a: Population at Risk Greater than a Given Survey Respondent

First let $Total.Risk(x) = Total.Risk_i$ for $i = x$ where $Total.Risk_i$ is as in Eq 5. And $PopRiskGreater(x)$ be the population represented by survey respondents who have risk greater than respondent x. P_i is as in Eq 6

$$PopRiskGreater(x) = \sum_{i=1}^{N.respondents} P_i * \begin{Bmatrix} 1 \ if \ Total.Risk_i > Total.Risk(x) \\ 0 \text{ otherwise} \end{Bmatrix}$$

We can then define $\boldsymbol{PopRiskGreater_i}$ to be $PopRiskGreater(x) \ for \ x = i$.

We then consider that at least some portion of the US population denoted as $P_{PrEP.Candidates}$ will have HIV risk sufficiently high to be a candidate for PrEP within the framework of our model. Intuitively, we can imagine that in our theoretical case where everyone in the US population is ranked by HIV risk, every xth person of the $Potential.PrEP.Population$ highest risk people will get PrEP (the PrEP supply will have been exhausted by the time individuals with risk lower than the $P_{PrEP.Candidates}$ are considered; in this case x=1÷$\lambda_{Efficiency}$).

Within this framework $P_{PrEP.Candidates}$ will equal the total PrEP prescriptions (N_{PrEP}) divided by the distribution efficiency. For example if there are one million prescriptions and $\lambda_{Efficiency}$ is 0.5, $P_{PrEP.Candidates}$ will equal two million since half of the top two million highest risk people will get PrEP for a total of one million prescriptions. Mathematically, this is shown as Eq 8b.

Eq 8b: Population of Potential PrEP Candidates

$$P_{PrEP.Candidates} = \frac{N_{PrEP}}{\lambda_{Efficiency}}$$

Now, we consider, how many people in the population corresponding to survey respondent $\boldsymbol{i}$ will receive PrEP as in **Table S3.**

Table S3: Number Of People In The Population Represented By Survey Respondent $\boldsymbol{i}$ Who Will Receive Prep

Case	Explanation	Mathematical Condition	$\boldsymbol{PrEP_i}$ *
1	Population represented by survey respondent $\boldsymbol{i}$ of insufficient risk to get PrEP	$PopRiskGreater_i > P_{PrEP.Candidates}$	0
2	Population represented by survey respondent $\boldsymbol{i}$ of sufficient risk to get PrEP and ample PrEP prescriptions remaining to cover the entire represented population represents (times the distribution efficiency)	$P_{PrEP.Candidates} \geq PopRiskGreater_i + P_i$	$\lambda_{Efficiency} * P_i$
3	Marginal case where "remaining" PrEP supply is only sufficient to cover a portion of the population covered by respondent $\boldsymbol{i}$	$PopRiskGreater_i + P_i > P_{PrEP.Candidates} > PopRiskGreater_i$	$N_{PrEP} - PopRiskGreater_i * \lambda_{Efficiency}$
*** $\boldsymbol{PrEP_i}$ indicates the number of PrEP users in the population represented by survey respondent $\boldsymbol{i}$**			

As defined in Table S3, $PrEP_i$ is the number of people in the population represented by survey respondent $\boldsymbol{i}$ on PrEP. $\lambda_{Efficiency}$ and N_{PrEP} in **Table S3** are as above and $\boldsymbol{P_i}$ is as in Eq 6. Intuitive, here, no PrEP is given if the supply has been exhausted by higher risk people (case 1). Most other individuals (case 2) are eligible for PrEP but only get it according to the distribution efficiency. Case 3 represents the marginal case of the one survey respondent where the represented population gets some PrEP but at a reduced rate because that respondent's population exhausts the supply. In case 3, $PopRiskGreater_i * \lambda_{Efficiency}$ will equal PrEP distributed to higher risk individuals; thus $\mathrm{N}_{PrEP} - PopRiskGreater_i * \lambda_{Efficiency}$ will represent the PrEP remaining for the population corresponding to the one survey respondent falling into case 3.

Using calculations for $\boldsymbol{PrEP_i}$ as in Table S3, we can calculate total HIV cases avoided in the population as in Eq 9. Intuitively, this is the PrEP risk of a respondent times the population to which the respondent scales times the proportion of individuals represented by the survey respondent on PrEP

Eq 9: Total HIV Cases Avoided by Survey Respondent

$$Cases.Avoided_i = PrEP_i * Total.Risk_i * Efficacy$$

Where $Cases.Avoided_i$ is the number of HIV cases avoided through PrEP in the population that scales from respondent $\boldsymbol{i}$**,** P_i is per Eq 6, $PrEP_i$ is per Eq 8 and $Total.Risk_i$ is per Eq 5. Intuitively, since $Total.Risk_i$ proportion of the population represented by survey respondent $\boldsymbol{i}$ would be expected to contract HIV in the absence of PrEP, multiplying this by the number of people on PrEP (as shown in Eq 9) will equal the cases avoided. $Efficacy$ is treated as a constant across all PrEP users representing the efficacy of PrEP, where we define efficacy as the average HIV relative risk reduction provided by PrEP use. In most analyses, we assume 100% efficacy but test this assumption in a supplemental analysis. As can be seen in this equation, the cases avoided will be directly proportional to one minus the efficacy.

Finally, we can sum over all respondents to find a final tally of cases avoided as in Eq 10.

Eq 10: Total HIV Cases Avoided Across the Population

$$Cases.Avoided.Total = \sum_{i=1}^{N.respondents} Cases.Avoided_i$$

where $Cases.Avoided.Total$ is the total cases avoided and $Cases.Avoided_i$ is per Eq 9; $N.respondents$ represents total survey respondents.

Parameter Values

Our default models assume that:

i. $\lambda_{Encounter\_Heterogenity}$ is arbitrarily small (encounters spread evenly across partners)
ii. No correlation between partner likelihood of STI and partner likelihood of HIV (λ_{phi}=0; see supplement)
iii. No correlation between respondent sexual activity with the partner and the partner's HIV likelihood ($\lambda_{BehaviorRisk} = 0$)
iv. 10% of heterosexual encounters involve anal intercourse in cases where the respondent indicates and history of anal intercourse. ($\lambda_{HAA\_Anal} = 0.1$)
v. Respondents engaging in homosexual anal intercourse have insertive intercourse on 50% of encounters and receptive intercourse on the other 50%
vi. assume partners the same age as the respondents

When not otherwise stated, we use these parameters. We vary assumptions i-vi individually in sensitivity analysis.

US Population at Risk and Total Sexually Transmitted HIV Cases

We estimated the US population aged 13-64 from US Census Bureau Data. The dataset we used listed population by age in bands of five years. We summed the population 15-64 directly and added 2/5 of the population 10-14 (to account for 13 and 14 year olds). We used 2024 county-level population by age and sex (summing by counties to get state-level data) (available at https://www2.census.gov/programs-surveys/popest/datasets/2020-2024/counties/asrh/cc-est2024-agesex-all.csv). We used these population data both to derive $P_{overall}$ and to sample US states for NHANES survey respondents.

We calculated overall HIV incidence due to sexual transmission from CDC data available at https://gis.cdc.gov/grasp/nchhstpatlas/tables.html. We used this overall incidence for calibration ($\lambda_{Status_{Adjustor}}$).

Specifically, we used 2022 HIV incidence by mode of transmission from the above URL. This indicates that there were approximately 28,400 new HIV cases in 2022 due to sexual transmission. However, approximately 1.6% of HIV cases (due to sexual and other modes of transmission occur in individuals 65 and over); this figure is based on 2022 HIV incidence by age from the CDC (https://gis.cdc.gov/grasp/nchhstpatlas/tables.html). Since our model excludes cases in those 65 or older, we scaled the total number of sexual transmissions down accordingly. This assumes that the proportion of HIV cases due to sexual transmission is the same in those over vs under 65; while this assumption is likely imperfect, given the very small percentage of total cases in individuals 65 and older, even significant deviations from this assumption would not materially impact our overall results. The final value of $\lambda_{Status_{Adjustor}}$ was 27,936.

Of note, the NHANES survey data used was based on individuals 18-59. We apply this to the population aged 13-65. The underlying assumption here is that sexual behavior and HIV transmission among individuals 18-59 offers a reasonable approximation for the slightly wider 13–65-year-old population. While this is likely imperfect, given that the 18–59-year older population encompasses most of the 13-65 population, deviations from these assumptions are likely have relatively minor impacts.

Model Implementation

We implemented models in R. We replicated the model 200 times for each set of parameters; with each replicate, we bootstrapped resampled (with replacement) survey respondents and re-imputed all stochastically imputed data elements. The parameter $\lambda_{Status_{Adjustor}}$ was fit using the optimizer package. All final results represent the median across the 200 replicates (with intervals representing the 10th-90th percentiles). Plots were generated using the ggplot2 package.

Additional Method Details

Calculating Distribution of Encounters across Partners

- While we can extract the number of male and female partners for each respondent and the total sexual encounters, we do not know the distribution of encounters between partners.
- For example, if a respondent had 30 encounters and 3 partners this could mean the respondent and one partner had 28 encounters and the two other partners with just 1 each; all three partners with 10 encounters or something in between.
- Our default is to assume that encounters are as evenly distributed as possible. (E.g. in the example above, each partner would have 10 encounters assigned).
- As a sensitivity analysis, we consider the other extreme where all partners except 1 have only 1 encounter each with the respondent. All remaining encounters are assigned to a single partner.
- We can generalize this to the following algorithm for assigning encounters to partners
  i. For x encounters across y partners (x≥y).
  ii. First assign each partner 1 encounter (by definition, each partner has to have 1 or more encounters)
  iii. Remaining encounters (z) equals x-y
  iv. Sequentially assign remaining z encounters by sampling partners for each encounter such that the probability of picking a given partner j is proportional to a the proportion of encounters previously assigned that were assigned to partner j raised to the $\lambda_{Encounter\_Heterogenity}$ power (for a parameter $\lambda_{Encounter\_Heterogenity}$)

     Mathematically, if g(j) is the number of encounters assigned to partner j prior to the iteration, we can then sample partners for a next encounter such that $pr(J) = \frac{[g(J)]^{\lambda_{Encounter\_Heterogenity}}}{\sum_{j=1}^{Total\ Parners}[g(j)]^{\lambda_{Encounter\_Heterogenity}}}$, where $pr(J)$ is the probability of selecting partner J on that iteration.
     - If $\lambda_{Encounter\_Heterogenity}$ is large negative number, we will achieve our default distribution (nearly equal partner distribution)
     - If $\lambda_{Encounter\_Heterogenity}$ is large positive number, we will achieve the alternative scenario of all but one partner with just one encounter
  v. Not withstanding the above, we define $\lambda_{Encounter\_Heterogenity}$ of -100 to represent maximum homogeneity and $\lambda_{Encounter\_Heterogenity}$ of 100 to represent maximum heterogeneity
  vi. In rare cases in which the reported number of partners exceeded the reported number of encounters, we assumed the reported partner count was accurate and assigned each partner a single encounter

Sampling Partner Ages

Partners ages are not directly available. For our primary analysis, we assume respondents and partners are the same age. For sensitivity analysis, we sample each partners' age by assuming a

differences in age between respondents and partners follow a normal distribution with a mean of 0 and a standard deviation of 5 years.

Estimating Likelihood of Each Partner HIV/STI Status

The starting point for assessing the likelihood of each partner HIV and STI status is the prevalence of HIV and STIs among the partner's age, sex and US State (later adjusted as in Eq 4a). We extracted this prevalence data from the CDC Atlas Plus Tool (https://gis.cdc.gov/grasp/nchhstpatlas/tables.html). We specifically used 2023 data.

We estimated the prevalence of acute HIV per 100,000 population as the number of reported HIV diagnoses per 100,000 (for the state and sex and age range) times (45/365), under the rough assumption that an individual has acute HIV for approximately 45 days on average. While this number is arbitrary, even varying it by a factor of several is unlikely to meaningfully impact the results since the rate of non-acute HIV is much higher than the rate of acute HIV.

We estimated the prevalence of non-acute as the reported HIV prevalence (for the age, sex and state) minus the estimated acute HIV prevalence. We multiplied this number by one minus the reported rate of viral load suppression (for the age, sex and state) to estimate the overall proportion with non-acute HIV capable of transmitting the virus to partners.

We took the *Chlamydia trachomatis* prevalence as the rate of "STI" for the purposes of this analysis. (We similarly used this rate when sampling respondent STI statuses).

Because HIV and STI prevalence are reported individually, we make several assumptions about the correlation at an individual level between having HIV and an STI. Our default assumption is no correlation (which is certainly incorrect, but offers and starting point). In this case, for example, we can estimate the prevalence of HIV without STI as the prevalence of HIV time 1 minus the prevalence of STI; likewise the prevalence of STI and HIV will equal the product of the prevalence of HIV and STI. (For STI prevalence, we use specifically the prevalence of *Chlamydia trachomatis*).

In sensitivity analysis, we introduce a parameter λ_{phi} representing the correlation (at an individual level) between HIV and STI prevalence. We then calculate the proportion of individuals co-infected with HIV and STI (and HIV-alone, STI-alone and neither infection) such that the correlation between having HIV and having an STI is λ_{phi} and the overall prevalence of HIV and of STI matches the CDC's reported prevalence. We transform prevalence (treated as a probability) to odds for use in Eq 4 using standard conversions between probabilities and odds (the baseline prevalence expressed as an odds is $prev.odds_{i,j,s}$ in these equations).

Sensitivity Analysis Evaluating The Impact Of Localized Variation In HIV Prevalence

Our primary framework uses HIV prevalence among individuals of the sexual partners' age, sex and US state as the baseline in estimating partner HIV risk (which is then adjusted by the calibration parameter and in some cases by a factor related in the survey respondent's sexual behavior as detailed above). However, this fails to account for potential local geographic areas or social networks with higher HIV prevalence.

We thus explored a series of additional scenarios:

i. all individuals live in the state with the lowest HIV prevalence for the partners age and sex ("low prevalence" scenario)
ii. all individuals live in the state with the highest HIV prevalence for the partners age and sex ("high prevalence" scenario)
iii. all individuals live in the state with the median HIV prevalence for the partners age and sex ("median prevalence with no variation" scenario)
iv. half of all partners are in a subgroup with an HIV prevalence equal to twice the median (by state per scenario iii) and half are in a subgroup with the prevalence half the median ("median prevalence with moderate variation" scenario)
v. 90% of all partners are in a subgroup with an HIV prevalence equal to one tenth the median and 10% are in a subgroup with the prevalence 10 times the median ("median prevalence with high variation" scenario)

We tested each scenario with default parameters (including lambda_behavior risk=0) and with lambda_behavior risk=1 (and otherwise default parameters).

SUPPLEMENTAL RESULTS

Table S4: Predicted Number of PrEP Users by Group as a Function of HIV Reduction Goal

		Predicted Number of PrEP Users 10th-90th percentiles across runs			
**Sample and PrEP Adoption**	**Distribution Efficiency**	**Female HAA**	**Male HAA**	**MSM**	**Total**
**Sampled Based on Survey Weights**					
PrEP Adoption to Achieve **a 25% reduction** in HIV Transmission	**Optimal (100%)**	2,528,000-3,821,000	0-0	0-303,000	2,622,000-3,859,000
	**50%**	4,753,000-6,018,000	0-146,000	30,000-490,000	4,976,000-6,395,000
PrEP Adoption to Achieve **a 50% reduction** in HIV Transmission	**Optimal (100%)**	9,507,000-12,035,000	0-292,000	60,000-979,000	9,952,000-12,789,000
	**50%**	NA	NA	NA	NA
PrEP Adoption to Achieve an **80% reduction** in HIV Transmission	**Optimal (100%)**	32,083,000-36,479,000	1,924,000-3,556,000	871,000-2,322,000	36,341,000-40,987,000
	**50%**	NA	NA	NA	NA
**MSM Upsampled by a Factor of Two**					
PrEP Adoption to Achieve a **25% reduction** in HIV Transmission	**Optimal (100%)**	2,487,000-3,776,000	0-0	0-598,000	2,618,000-4,011,000
	**50%**	4,699,000-5,919,000	0-144,000	59,000-964,000	5,066,000-6,710,000
PrEP Adoption to Achieve a **50% reduction** in HIV Transmission	**Optimal (100%)**	9,398,000-11,837,000	0-289,000	119,000-1,927,000	10,132,000-13,420,000
	**50%**	NA	NA	NA	NA
PrEP Adoption to Achieve an **80% reduction** in HIV Transmission	**Optimal (100%)**	31,689,000-36,027,000	1,887,000-3,507,000	1,732,000-4,569,000	37,252,000-42,135,000
	**50%**	NA	NA	NA	NA

Shown are the estimated numbers (ranges represent 10th-90th percentiles across the 200 simulations) for the number of individuals within each group who may need PrEP at optimal and 50% distribution efficiency to reduce HIV transmission by 25%, 50% and 80%. Because MSM individuals may have been undersampled in the NHANES survey, we performed a secondary analysis in which we upweighted MSM individuals by a factor of two (bottom set of rows). Because values are subject to the assumptions and limitations of the overall model, results should be considered in the appropriate context and alongside other available evidence.

Table S5: Illustrative Examples of Individuals Who the Model Predicts May Need PrEP

Individual	Description	On PrEP
1	**57 year old Female (HAA) in TX**<br>**1 Male Partner**<br>Anal Receptive with Condom | 31 times,<br>Vaginal Receptive with Condom | 203 times	50% Reduction Target
2	**44 year old Female (HAA) in TX**<br>**1 Male Partner**<br>Anal Receptive with Condom | 5 times,<br>Vaginal Receptive with Condom | 27 times	80% Reduction Target
3	**34 year old Female (HAA) in IL**<br>**3 Male Partners**<br><br>Partner 1:<br>Anal Receptive with Condom | 1 times,<br>Anal Receptive without Condom | 1 times,<br>Vaginal Receptive with Condom | 7 times,<br>Vaginal Receptive without Condom | 17 times<br>Partner 2:<br>Anal Receptive with Condom | 1 times,<br>Anal Receptive without Condom | 1 times,<br>Vaginal Receptive with Condom | 5 times,<br>Vaginal Receptive without Condom | 19 times<br>Partner 3:<br>Anal Receptive without Condom | 2 times,<br>Vaginal Receptive with Condom | 3 times,<br>Vaginal Receptive without Condom | 21 times	50% Reduction Target
5	**25 year old Male (MSM) in NC**<br>**3 Male Partners**<br><br>Partner 1:<br>Anal Penetrative with Condom | 6 times,<br>Anal Receptive with Condom | 5 times<br>Partner 2:<br>Anal Penetrative with Condom | 3 times,<br>Anal Receptive with Condom | 8 times<br>Partner 3:<br>Anal Penetrative with Condom | 5 times,<br>Anal Receptive with Condom | 5 times	80% Reduction Target
6	**26 year old Male (HAA) in NY**<br>**2 Female Partners**<br><br>Partner 1:<br>Anal Penetrative without Condom | 28 times,<br>Vaginal Penetrative without Condom | 246 times<br>Partner 2:<br>Anal Penetrative without Condom | 28 times,<br>Vaginal Penetrative without Condom | 246 times	80% Reduction Target

Shown are five illustrative examples of survey respondents from a single model run (run not included in the main analysis). Each row shows the imputed sexual behavior and US states for the model run for the survey respondent along with the model's HIV risk prediction and whether the model (for the single run) would expect this individual to be on PrEP if PrEP were targeted to achieve a 50% of or an 80% reduction in HIV transmission. Given the model assumptions and the fact that these are from a single run, these examples are for illustrative purposes only and should not be used to inform exact risk assessment.

Figure S1: Population HIV Reduction vs. Individual Risk Threshold at Which PrEP is Given

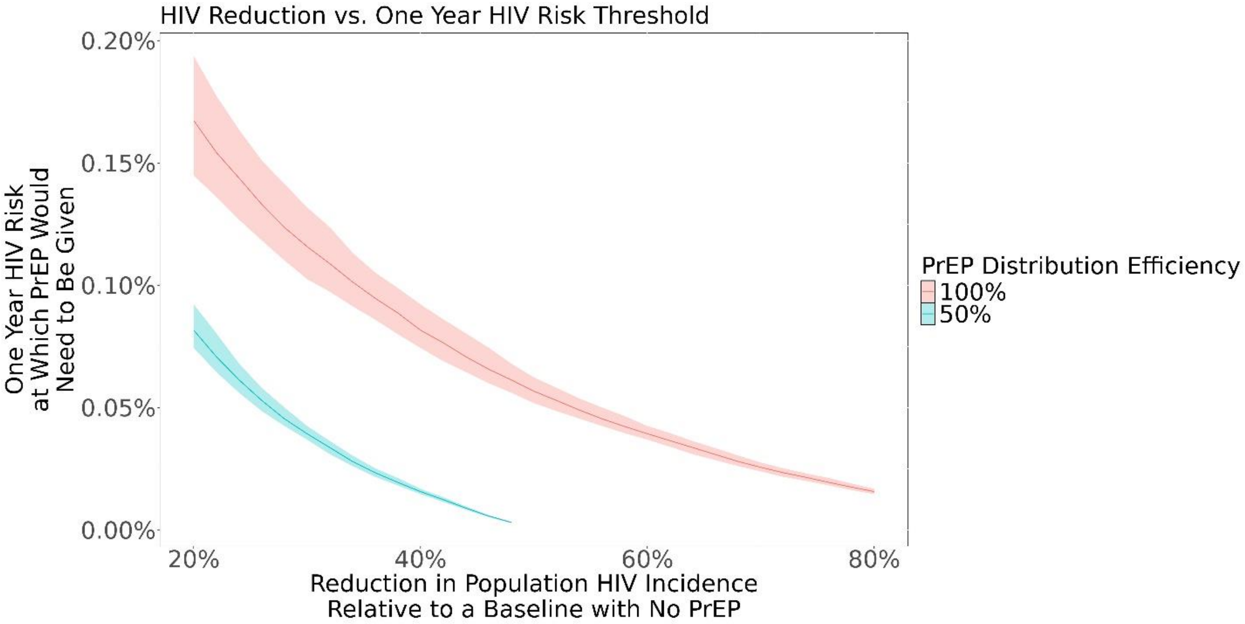

Plotted is the annualized HIV risk threshold above which individuals would need to be on PrEP (y-axis) to reduce sexually transmitted HIV incidence by the percentage (from baseline) shown on the x-axis. For example, under 100% distribution efficiency, to reduce HIV transmission by 50%, individuals with an annual HIV risk of greater than about 0.06% would need to be given PrEP. These risk thresholds are considerably lower than the 1% annualized risk other sources have used to evaluate PrEP need. This suggests that i) to achieve HIV elimination goals, PrEP may need to be offered to individuals at lower annualized HIV risk than some prior estimates and ii) our model is likely suboptimally calibrated. The suboptimal calibration likely stems from some of the limitations noted in the main manuscript, particularly the over-homogenization of HIV risk among the partners of survey respondents. Nonetheless, it may be important to recognize that only offering PrEP to individuals with a risk greater than 1% may fail to reach HIV reduction goals, recognizing that if PrEP is offered to individuals with too low an individual risk, the PrEP risks may outweigh the benefits at the level of the individual PrEP user.

Figure S2: Number Needed to Treat as a Function of PrEP Adoption and Distribution Efficiency

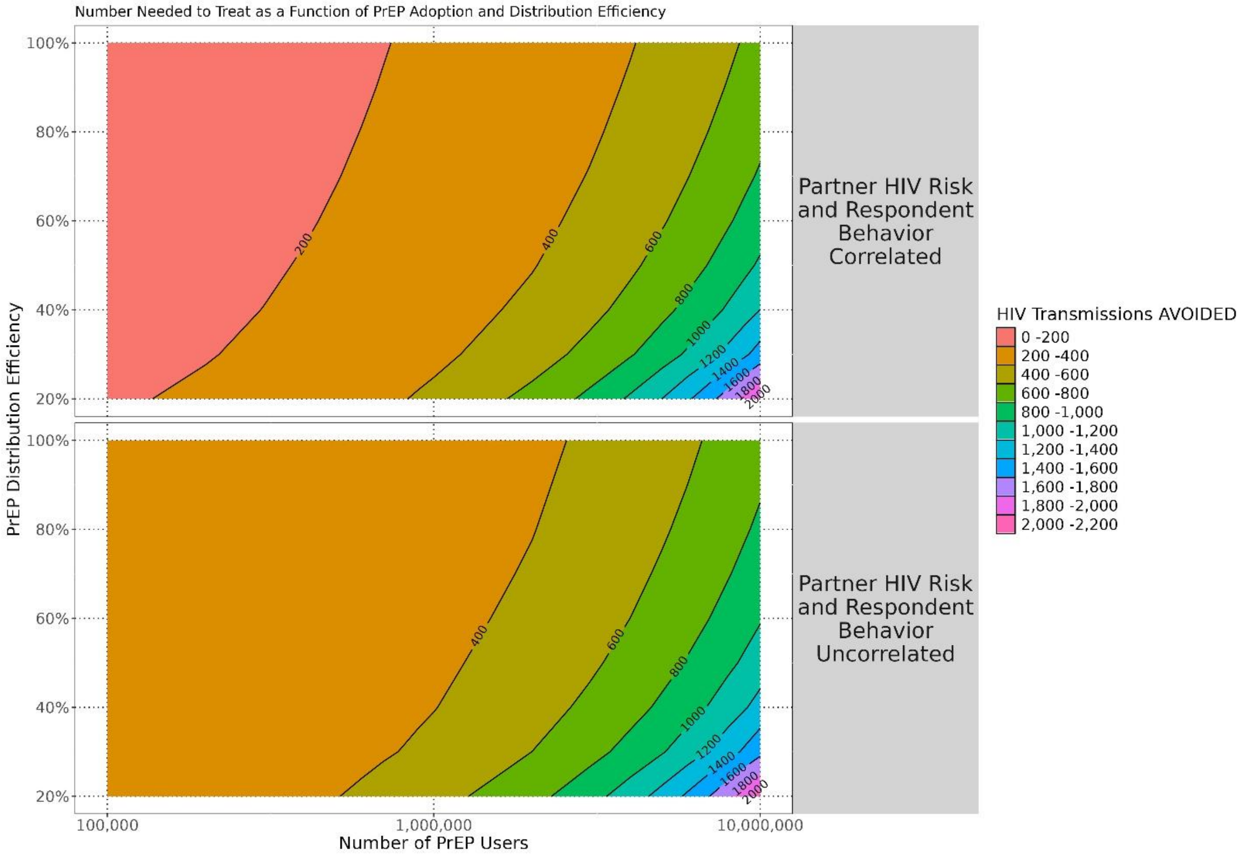

Shown is the number needed to treat (i.e., number of individuals on PrEP per HIV case avoided) as function of PrEP adoption (x-axis) and distribution efficiency (y-axis). Color and contours describe number needed to treat.

Figure S3: Impact of Imperfect PrEP Efficacy

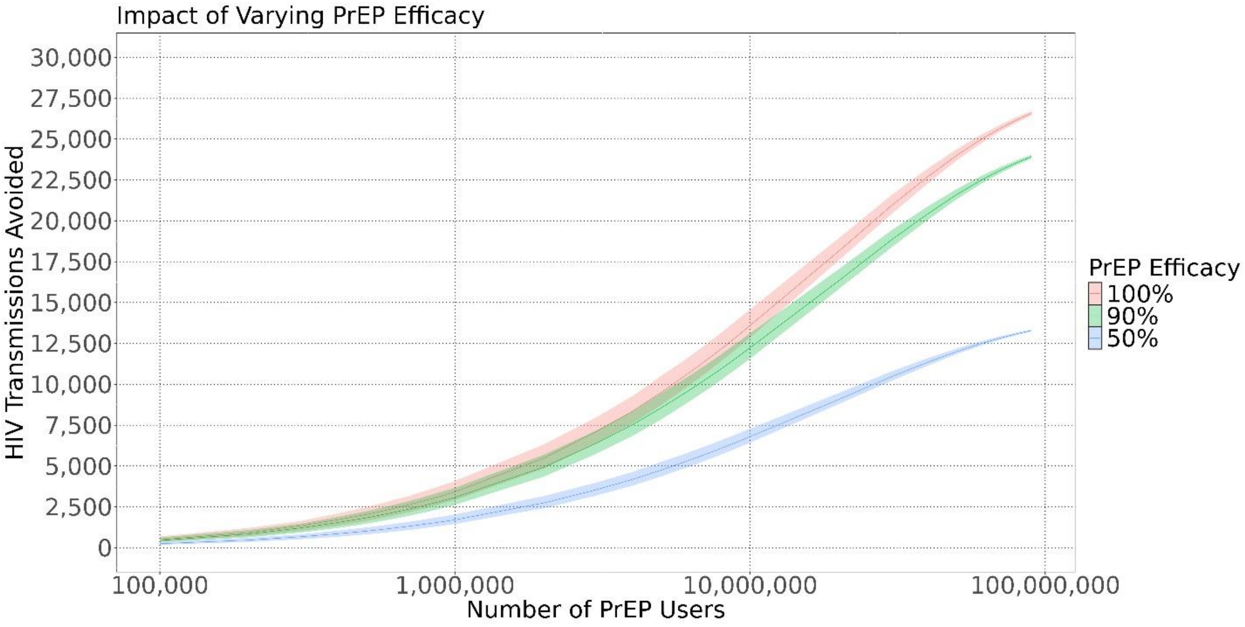

Shown are the number of HIV transmissions avoided as a function of the number of PrEP users, assuming optimal PrEP distribution efficiency and no correlation between partner HIV risk and respondent sexual behavior. The red curve (PrEP Efficacy 100%) is equivalent to our primary analysis (with perfect distribution efficiency and no correlation between partner HIV risk and respondent sexual behavior). The other curves, representing 90% and 50% PrEP efficacy show a proportional decrease in cases avoided (cases avoided is proportional to one minus the PrEP Efficacy).

Figure S4: Sensitivity Analysis Examining the Impact of Introducing Correlation Between Partner HIV likelihood and STI likelihood

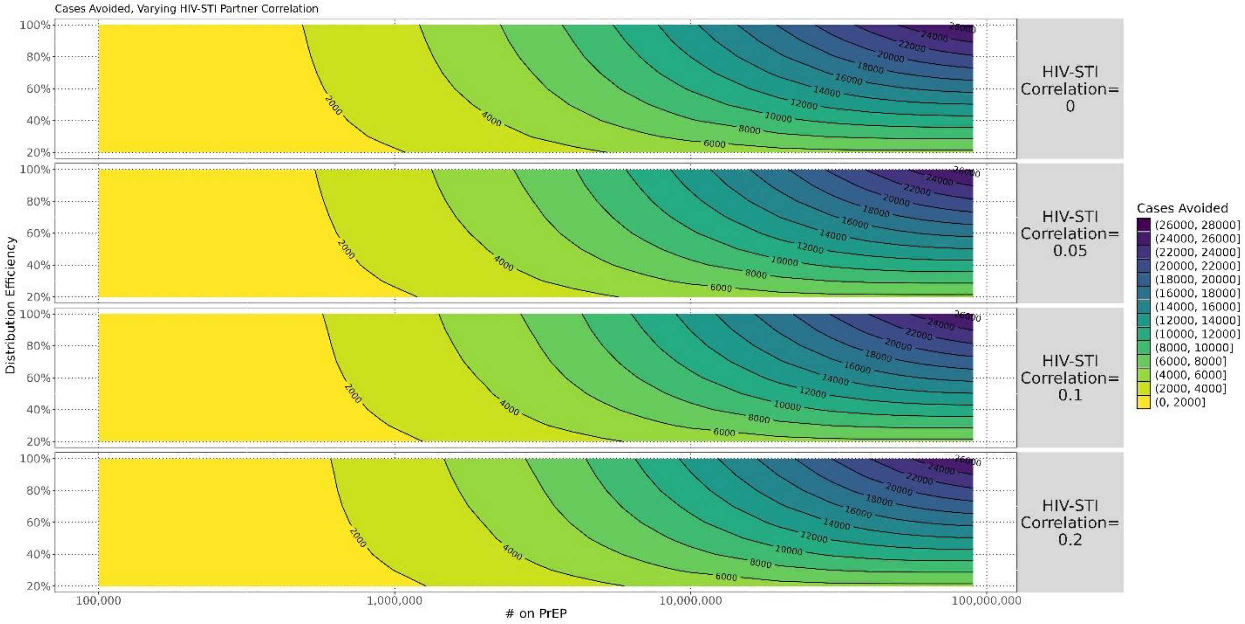

Shown is the effect on PrEP impact if HIV likelihood is correlated with STI likelihood within the sexual partners of survey respondents. The x-axis is PrEP adoption and y-axis is distribution efficiency, analogous to Figure 3. The top row (no correlation) represents our default case. Our model predicts PrEP to be slightly more effective at a given level of adoption and distribution efficiency if HIV and STI likelihood are more correlated as evidenced by the contours "shifting left" in lower rows in the table. Mathematically, the HIV-STI correlation represents λ_{phi} described above.

Figure S5: Sensitivity Analysis, Impact of varying rates of anal intercourse among HAA

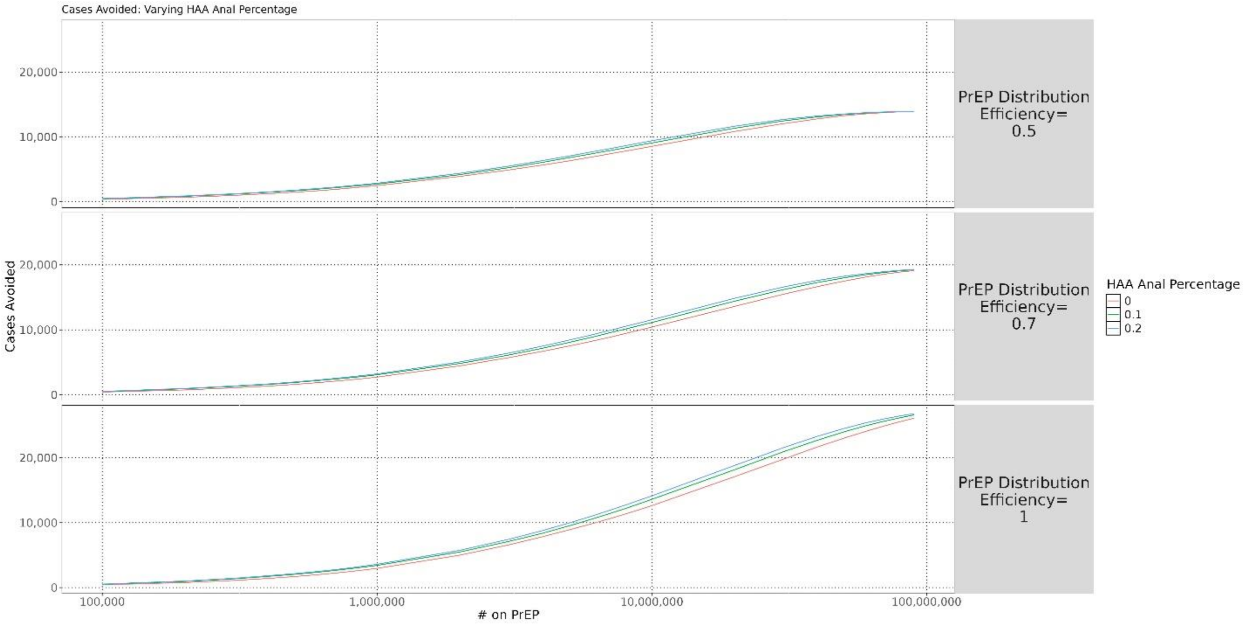

Shown is the effect on PrEP impact (cases avoided, y-axis) of varying the proportion of intercourse comprised of anal sex among heterosexual individuals who report having had anal sex (represented by color of line). The x-axis is PrEP adoption and panels represent varying distribution efficiencies. Varying the percentage of heterosexual intercourse comprised by anal sex has at most a very modest impact on model output. Mathematically, the percentages of anal sex correspond to λ_{HAA_Anal} in Table S1.

Figure S6: Sensitivity Analysis, Impact Of Varying Correlation Between Partner HIV-1 Infection Status And Respondent Sexual Risk Behavior

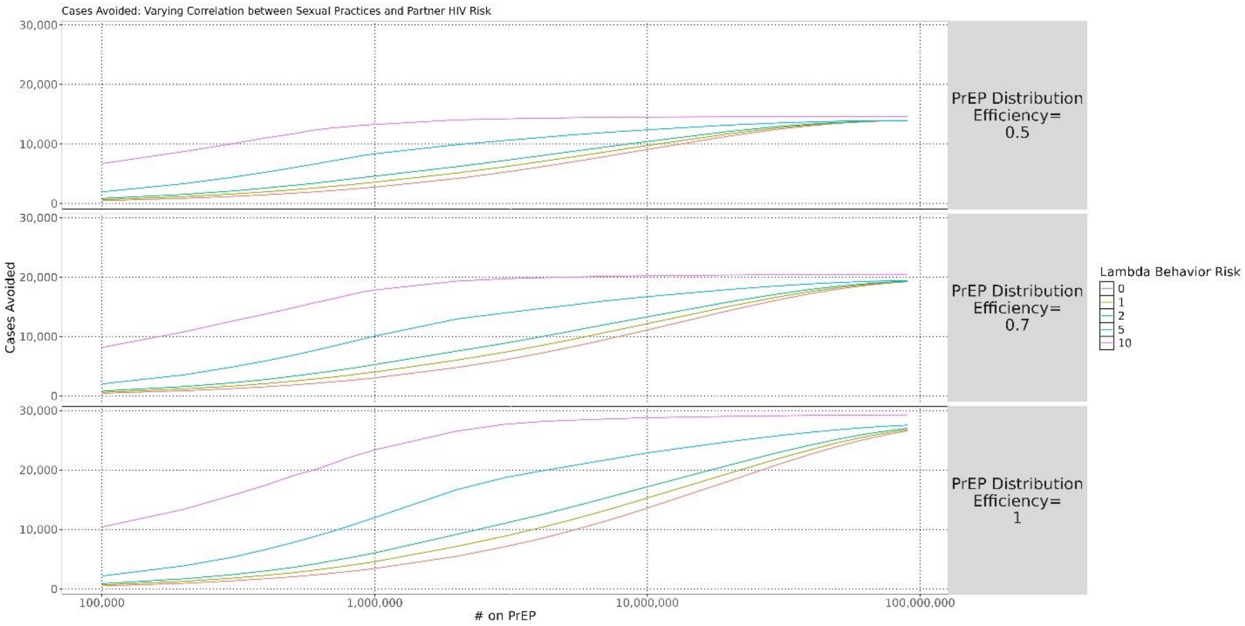

Shown is the effect on PrEP impact (cases avoided, y-axis) of varying the correlation between partner likelihood of having HIV and the survey respondent's sexual behavior (Lambda Behavior Risk, corresponding to $\lambda_{BehaviorRisk}$ $in\ Eq\ 4a$). The x-axis is PrEP adoption and panels represent varying distribution efficiencies.

If survey respondents who themselves engage in riskier sexual activity have partners who are more likely to have HIV (higher values of Lambda Behavior Risk) the model predicts PrEP will be more impactful (i.e., more HIV cases avoided at a given level of adoption and distribution efficiency). This is in comparison to our base case where partner HIV likelihood is independent of survey respondent sexual practices (i.e. $\lambda_{BehaviorRisk} = 0$ in our base case).

Figure S7:. Sensitivity Analysis, Impact Of Varying Distribution Of Sexual Encounters Across Sexual Partners

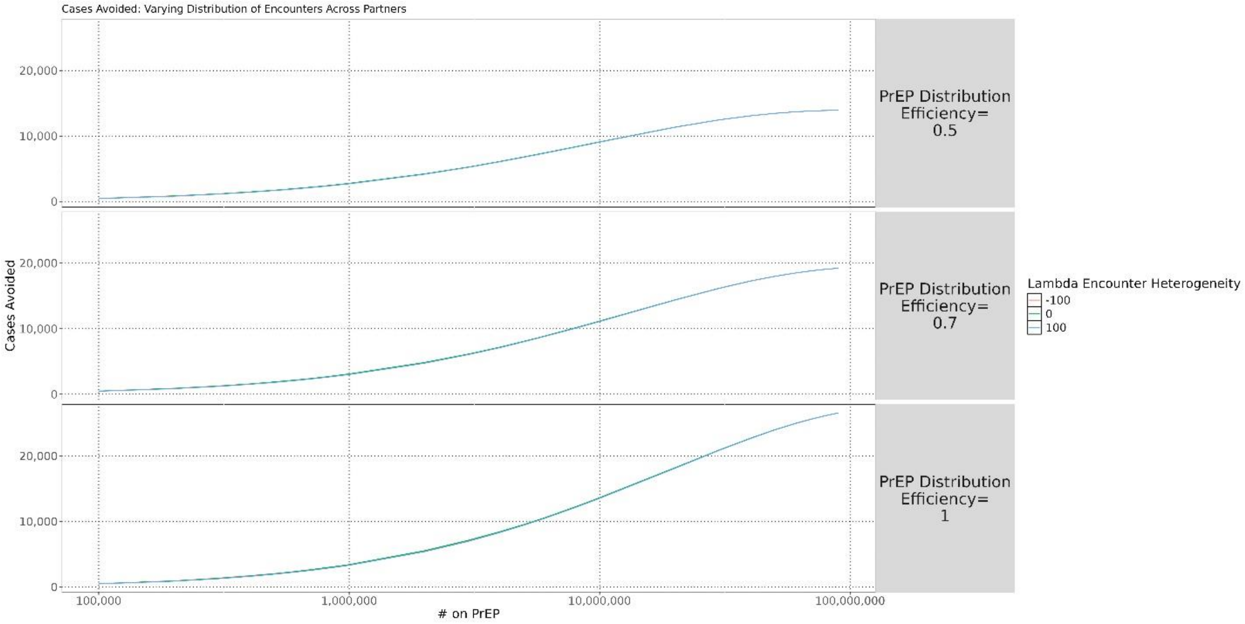

Shown is the effect on PrEP impact (cases avoided, y-axis) of varying distribution of a survey respondents sexual encounters across partners, for respondents who report multiple partners. x-axis is PrEP adoption and panels represent varying distribution efficiencies.

The distribution of encounters across partners is controlled by the parameter (Lambda Encounter Heterogeneity in the legend equivalent to $\lambda_{Encounter\_Heterogenity}$ in the section above on calculating the distribution encounters across partners). Lambda Encounter Heterogeneity of -100 is our base case in which encounters are spread as uniformly as possible across partners; Lambda Encounter Heterogeneity of 100 is the other extreme in which all but one partner have only one encounter each with the remaining sexual encounters attributed to a single partner. Lambda Encounter Heterogeneity of 0 is in between these extremes. Regardless, varying this parameter has no material impact on the results (curves above are overlapping and indistinguishable).

Figure S8: Sensitivity Analysis, Impact Of Varying Distribution Of Receptive Vs. Insertive Intercourse Among MSM Individuals

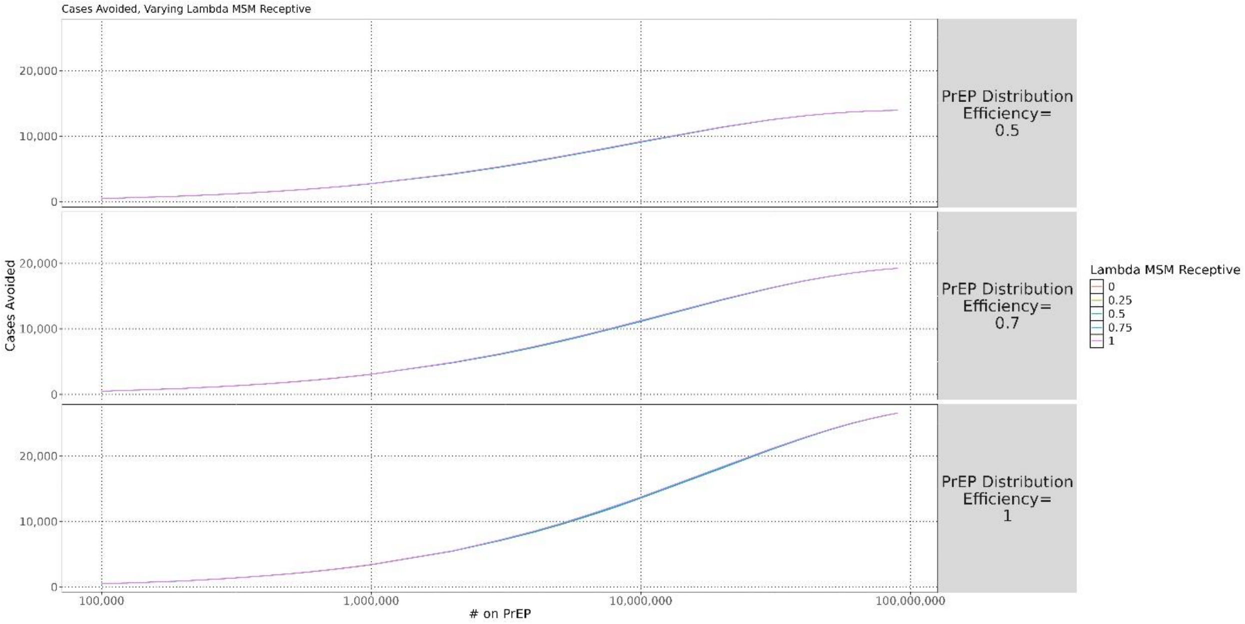

Shown is the effect on PrEP impact (cases avoided, y-axis) of varying distribution of receptive vs insertive intercourse for sexual encounters involving anal sex between two male individuals. x-axis is PrEP adoption and panels represent varying distribution efficiencies.

The distribution of receptive vs insertive intercourse is controlled by the parameter (Lambda MSM Receptive as listed in the legend equivalent to $\lambda_{\mathrm{MSM\_receptive}}$ in Table S1). Lambda MSM Receptive 0.5 is our base case in which a male individual engaging in homosexual anal intercourse with another male will have on average receptive intercourse 50% of the time and insertive 50% of the time. Likewise at a Lambda MSM Receptive 0.75 or 0.25, a given male will have insertive intercourse 25% or 75% of the time, respectively, with the remainder being receptive. At a value of 0 or 1, individuals will only have either insertive or receptive intercourse respectively.

Varying the value of this parameter has no material impact on results (curves above are overlapping and indistinguishable).

Figure S9: Sensitivity Analysis, Impact Of Varying Distribution Of Age Difference Between Survey Respondents And Their Partners

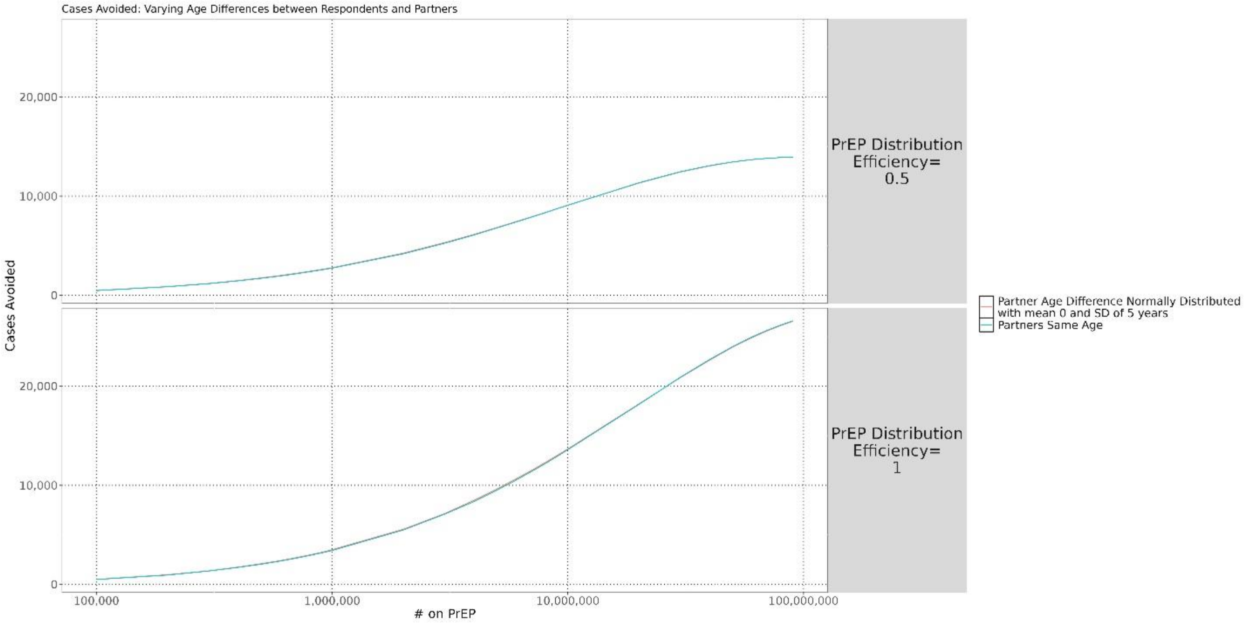

Shown is the effect on PrEP impact (cases avoided, y-axis) of varying the distribution of age differences between survey respondents and their partners. x-axis is PrEP adoption and panels represent varying distribution efficiencies. The base case is that the partners are the same age as the survey respondent. The alternative case shown here in which the respondents and partners vary in age according to a normal distribution with a mean of 0 and a standard deviation of five years produced no discernable impact on results (curves above are overlapping and indistinguishable).

Figure S10: Sensitivity Analysis, Impact Of Variation In HIV Prevalence Among Localized Subpopulations

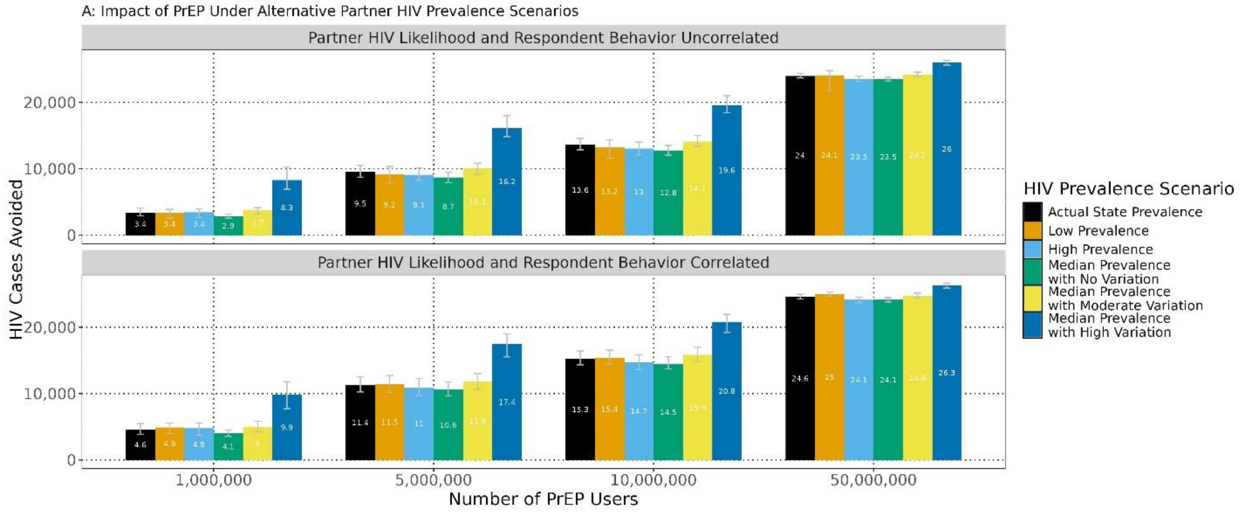

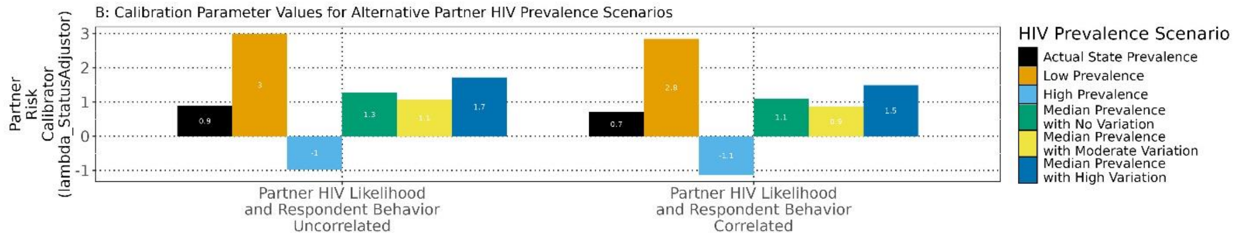

Shown in part A is the effect on PrEP impact (cases avoided, y-axis) of assuming that subpopulations with local patterns of admixture may have HIV prevalence that varies significantly from the state-wide prevalence levels used in our base case. The x-axis represents PrEP adoption. Results are shown for the case of 100% distribution efficiency.

Shown are six scenarios: **Actual State Prevalence**: original model; **Low prevalence**: all individuals live in the state with the lowest HIV prevalence for the partners age and sex; **High prevalence**: all individuals live in the state with the highest HIV prevalence for the partners age and sex; **Median prevalence with No Variation**: all individuals live in the state with the highest HIV prevalence for the partners age and sex; **Median prevalence with Moderate Variation**: half of all partners are in a subgroup with an HIV prevalence equal to twice the median and half are in a subgroup with the prevalence half the median; **Median prevalence with High Variation**: 90% of all partners are in a subgroup with an HIV prevalence equal to one tenth the median and 10% are in a subgroup with the prevalence 10 times the median. For each scenario, we estimated the PrEP impact at various levels of adoption and 100% distribution efficiency (Part A). Changing baseline prevalence (low vs medium vs high) had negligible impact on estimate PrEP impact; rather it just changed the calibration parameter to ensure proper model calibration to overall HIV incidence (Part B). However, increased variation in prevalence did have a

moderate impact on PrEP impact. lambda_StatusAdjustor (y-axis in Part B) is equivalent to $\lambda_{Status_{Adjustor}}$ in Eq 4a.
